# Susceptibility to type 2 diabetes may be modulated by haplotypes in *G6PC2*, a target of positive selection

**DOI:** 10.1186/s12862-017-0897-z

**Published:** 2017-02-07

**Authors:** Nasser M. Al-Daghri, Chiara Pontremoli, Rachele Cagliani, Diego Forni, Majed S. Alokail, Omar S. Al-Attas, Shaun Sabico, Stefania Riva, Mario Clerici, Manuela Sironi

**Affiliations:** 1 Biomarker research program, Biochemistry Department, College of Science, King Saud Universiy, Riyadh, 11451 Kingdom of Saudi Arabia; 2 Prince Mutaib Chair for Biomarkers of Osteoporosis Research, King Saud University, Riyadh, 11451 Kingdom of Saudi Arabia; 3 Scientific Institute IRCCS E.MEDEA, Bosisio Parini, 23842 Italy; 4 Department of Physiopathology and Transplantation, University of Milan, via F.lli Cervi 93, Segrate, 20090 Milan, Italy; 5 Don Gnocchi Foundation, ONLUS, Milan, 20148 Italy

**Keywords:** *G6PC2*, *G6PC*, *G6PC3*, Natural selection, Association analysis, Type 2 diabetes

## Abstract

**Background:**

The endoplasmic reticulum enzyme glucose-6-phosphatase catalyzes the common terminal reaction in the gluconeogenic/glycogenolytic pathways and plays a central role in glucose homeostasis. In most mammals, different G6PC subunits are encoded by three paralogous genes (*G6PC*, *G6PC2*, and *G6PC3*). Mutations in *G6PC* and *G6PC3* are responsible for human mendelian diseases, whereas variants in *G6PC2* are associated with fasting glucose (FG) levels.

**Results:**

We analyzed the evolutionary history of G6Pase genes. Results indicated that the three paralogs originated during early vertebrate evolution and that negative selection was the major force shaping diversity at these genes in mammals. Nonetheless, site-wise estimation of evolutionary rates at corresponding sites revealed weak correlations, suggesting that mammalian G6Pases have evolved different structural features over time. We also detected pervasive positive selection at mammalian *G6PC2*. Most selected residues localize in the C-terminal protein region, where several human variants associated with FG levels also map. This region was re-sequenced in ~560 subjects from Saudi Arabia, 185 of whom suffering from type 2 diabetes (T2D). The frequency of rare missense and nonsense variants was not significantly different in T2D and controls. Association analysis with two common missense variants (V219L and S342C) revealed a weak but significant association for both SNPs when analyses were conditioned on rs560887, previously identified in a GWAS for FG. Two haplotypes were significantly associated with T2D with an opposite effect direction.

**Conclusions:**

We detected pervasive positive selection at mammalian *G6PC2* genes and we suggest that distinct haplotypes at the *G6PC2* locus modulate susceptibility to T2D.

**Electronic supplementary material:**

The online version of this article (doi:10.1186/s12862-017-0897-z) contains supplementary material, which is available to authorized users.

## Background

The endoplasmic reticulum enzyme glucose-6-phosphatase catalyzes the hydrolysis of glucose-6-phosphate (G6P) to glucose and inorganic phosphate. The enzyme is part of a multicomponent integral membrane system that includes the catalytic subunit (G6PC, hereafter referred to as G6Pase) as well as transporters for glucose-6-phosphate, inorganic phosphate, and glucose [1, 2]. G6Pase catalyzes the common terminal reaction in the gluconeogenic and glycogenolytic pathways, resulting in the release of glucose into the bloodstream [1]. These results led to the identification of G6Pase as a key player in glucose homeostasis.

In most mammals, different G6PC subunits are encoded by three paralogous genes (*G6PC*, *G6PC2*, and *G6PC3*), usually referred to as the *G6PC* gene family [1, 2]. The protein products of the three genes display moderate sequence identity and a common topological organization with nine transmembrane domains and intralumenal catalytic residues [1].

G6PC is mainly expressed in the liver and kidney and at lower levels in the intestine and pancreatic islets, and has a critical function in maintaining euglycemia in fasting conditions [1, 2]. In humans, mutations in the gene cause glycogen storage disease type Ia (GSD1A), which results in severe hypoglycemia and glycogen accumulation-associated hepatomegaly, as well as growth retardation, lactic acidemia, hyperlipidemia, hyperuricemia, and increased incidence of hepatic adenomas [1, 2]. Mutations in *G6PC3* are also associated with pathology in humans. Thus, although the gene is ubiquitously expressed, its function is particularly important in white blood cells, and G6PC3 deficiency causes autosomal recessive severe congenital neutropenia type 4 (SCN4) [1, 2]. SCN4 patients are particularly susceptible to bacterial infections and may display additional non immunologic symptoms. Conversely, in both humans and in the knock-out mouse model, G6PC3 only marginally contributes to the regulation of blood glucose levels or hepatic glycogen content [1, 2]. Finally, G6PC2 is specifically expressed in pancreatic islets where its function is still incompletely understood [1, 2]. *g6pc2* ^*−/−*^ mice display a reduction in blood glucose levels after a 6 h fast, whereas plasma insulin and glucagon concentrations are unaffected [1, 2]. These data led to the hypothesis that G6PC2 regulates the glycolytic flux by hydrolyzing G6P, thereby opposing the action of glucokinase. G6PC2 and glucokinase are, therefore suggested to function as beta islet glucose sensors [1, 2]. In humans, common and rare variants in *G6PC2* have been associated with fasting glucose (FG) levels and with decreased insulin secretion during glucose tolerance tests [3–9]. This observation led to the suggestion that G6PC2 may also regulate the pulsatility of insulin secretion [1, 2].

Variation in FG is clinically important in humans, as it is associated with the risk of developing type 2 diabetes (T2D) and ischemic heart disease [10, 11] as well as being an important determinant of offspring birth weight in pregnant women [12].

In humans and other mammals, FG levels are influenced by the feeding status. Prolonged fasting causes a reduction in blood glucose levels, which can result in life-threatening hypoglycemia; the gluconeogenic pathway is the major contributor to the maintenance of glucose levels during fasting and starvation [13]. Mammals display a wide variety of diets, different lifestyles (that may or may not include recurrent prolonged fasts), and distinct energy requirements. These characteristics influence the ability of a species to resist prolonged fasting [13], a situation that is common in nature and that is likely to exert a strong selective pressure. It is thus conceivable that genes involved in the regulation of FG have been targeted by positive (or diversifying) selection during mammalian evolution. Indeed, positive selection was previously demonstrated to act on genes with a role in carbohydrate absorption and digestion in mammals [14, 15]. In humans, aside from the textbook example of lactase persistence [16], signals of diet-driven selection include variants in genes involved in starch and sucrose metabolism [15, 17], copy number variation at genes encoding salivary amylase (*AMY1*) [18], as well as polymorphisms in genes that may be associated with the consumption of cooked food [19]. In fact, humans likely underwent several dietary shifts associated with cultural innovations such as the use of fire for cooking (likely predating the split of modern humans from Neanderthals/Denisovans) [19, 20], the exploitation of starch-rich plant underground storage organs [21], and the agricultural revolution. Because these cultural changes modified diet composition and caloric intake, genes involved in glucose homeostasis, such as *G6PC* genes, represent likely target of positive selection in humans.

Herein we use both inter- and intra-species comparisons to analyze the evolution of the three G6Pase genes in mammals and human populations. We also perform an association study to assess the role of *G6PC2* variants in T2D susceptibility in a population with high incidence of metabolic disorders.

## Results

### Evolutionary origin of the *G6PC* gene family

We first investigated the evolutionary origin of the three mammalian *G6PC* paralogs. Analysis of a gene gain/loss tree of 70 animal species through the Ensembl Compara utility [22, 23] indicated that a single *G6PC* gene is present in the *Drosophila* genome, whereas lamprey (*Petromyzon marinus,* Cyclostomata) displays two genes and most bony fishes, birds, reptiles, amphibians and mammals have at least three paralogs. Possibly due to gene loss, no *G6PC* gene is described in the two Tunicata genomes included in the Ensembl Compara dataset.

**Fig. 1:**
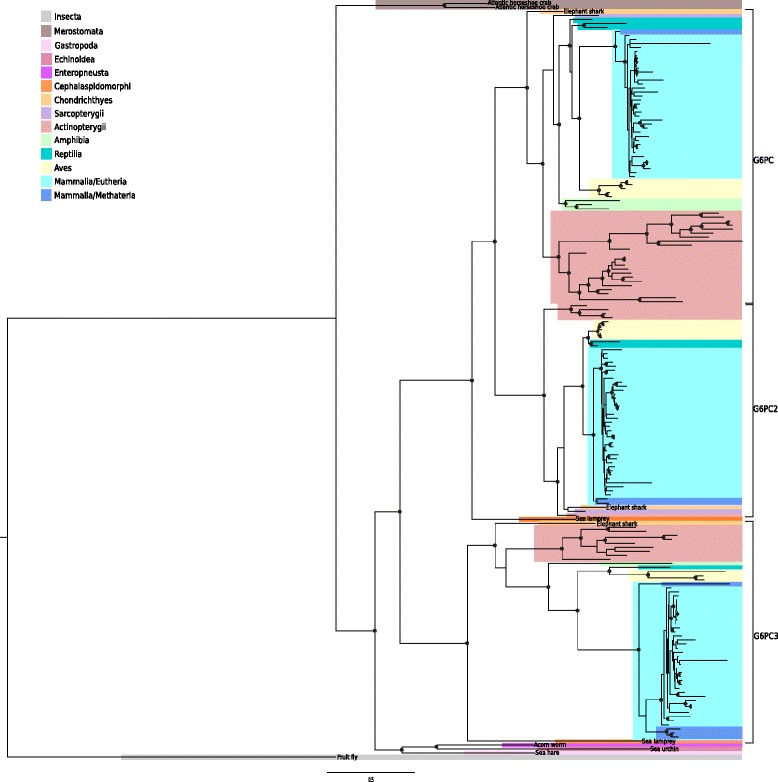
Maximum likelihood phylogenetic tree of metazoan G6PC proteins. Colored boxes indicate the class of each species (for a list of species see Additional file 1: Table S1), as reported in the legend. Black dots indicate bootstrap values greater than 50%

Overall, these observations suggest that the first duplication of an ancestral *G6PC* gene occurred during the vertebrate/invertebrate split and a second duplication took place either in the ancestor of all Gnathostomata (jawed vertebrates) or in the ancestor of bony vertebrates (i.e. after the split of bony and cartilaginous fishes). To more precisely map these duplication events, we constructed a phylogenetic tree using protein sequence information for the animal species included in the Ensembl database plus additional organisms selected to resolve the timing of the duplication events (Fig. 1, Additional file 1: Table S1). Results indicated that arthropods, mollusks, and echinoderms display one single *G6PC* gene, with the only exception of *Limulus polyphemus*, which has two highly similar genes suggesting a recent duplication event in this lineage. One *G6PC* gene is also observed in the hemichordate *Saccoglossus kowalevskii*. No *G6PC* gene was identified in the genomes of tunicates and cephalochordates, suggesting lineage-specific losses.

Analysis of the *G6PC* phylogeny indicated that an initial duplication event in the lineage basal to all vertebrates originated *G6PC3* and the *G6PC/G6PC2* ancestor. In lamprey, one of the two G6PC sequences clusters with G6PC3 proteins, whereas the other is basal to G6PC2 and G6PC (Fig. 1), suggesting that the duplication events that originated *G6PC* and *G6PC2* occurred after the split of gnathostomes and cyclostomes but before the divergence of cartilaginous and bony fishes, as the three *Callorhinchus milii* sequences (the elephant shark) indicate (Fig. 1).

### Evolutionary analysis of the glucose-6-phosphatase (G6PC) catalytic subunit gene family in mammals

We next analyzed in detail the evolutionary history of the three genes encoding G6Pases in eutherian mammals. To this aim, coding sequence information for ~64 species were retrieved (Table 1 and Additional file 1: Table S2). Specifically, all available sequences with good coverage were retrieved for the study. The rat sequence was not included for *G6PC2,* as the gene is non functional in this rodent species [24]. GARD (genetic algorithm recombination detection) [25] detected no recombination breakpoint in any alignment. To obtain an estimate of the extent of functional constraint acting on these genes, we calculated the average non-synonymous substitution/synonymous substitution rate (dN/dS, also referred to as ω) using the single-likelihood ancestor counting (SLAC) method [26]. As is the case for most mammalian genes [27], dN/dS was always lower than 1 (Table 1), indicating that purifying selection is the major force shaping diversity at mammalian G6Pase genes. Indeed, analysis based on the fixed effects likelihood (FEL) method [26] detected a considerable proportion of negatively selected sites in all three genes (Table 1). The protein products of the three genes share a common topological structure, display considerable sequence identity, and perform the same molecular function, albeit in different cell types. To test whether structural/functional constraints represent major drivers of molecular evolution, we used FEL to calculate the normalized dN-dS value at each site and we correlated this parameter across corresponding sites (on the basis of the pairwise protein alignments). Although a significant correlation between dN-dS values was detected for *G6PC* and *G6PC2* (Spearman’s rank correlation, *p* = 0.0062), as well as for *G6PC* and *G6PC3* (Spearman’s rank correlation, *p* = 0.0025), the correlation coefficients were small (ρ = 0.15 and 0.16, respectively). No significant correlation was detected for the *G6PC2*-*G6PC3* pair (Spearman’s rank correlation, *p* = 0.123, ρ = 0.08).

**Table 1:** Average non-synonymous/synonymous substitution rate ratio (dN/dS) and percentage of negatively selected sites fot the three G6Pase genes

Gene	ALIAS	Protein size (amino acids)	Tree Lenght	N° of species	Average dN/dS (95% confidence intervals)	% of FEL negatively selected sites
*G6PC*	*G6PT*	357	8.65	64	0.167 (0.156, 0.178)	66.39%
*G6PC2*	*IGRP*	355	6.44	64	0.206 (0.191, 0.222)	52.11%
*G6PC3*	*UGRP*	346	7.62	62	0.159 (0.147, 0.171)	66.18%

A common expectation is that mutations at highly constrained codons are more likely to disrupt protein structure/function and, therefore, to cause disease. To date, 57 independent GSD1A missense mutations involving 47 unique codons have been reported. We observed that codons that carry at least one missense mutation are significantly more likely to show statistical evidence of negative selection (FEL *p* value < 0.1) than codons where no mutation has been described (Fisher’s Exact Test, two tailed, *p* = 0.044, odds ratio = 2.19, 95% confidence intervals: 0.99–5.34). The same calculation was not performed for *G6PC3* mutations, as too few of such mutations are actually known (number of mutated codons = 9, seven of which negatively selected).

### Positive selection at the mammalian *G6PC2* gene

**Fig. 2:**
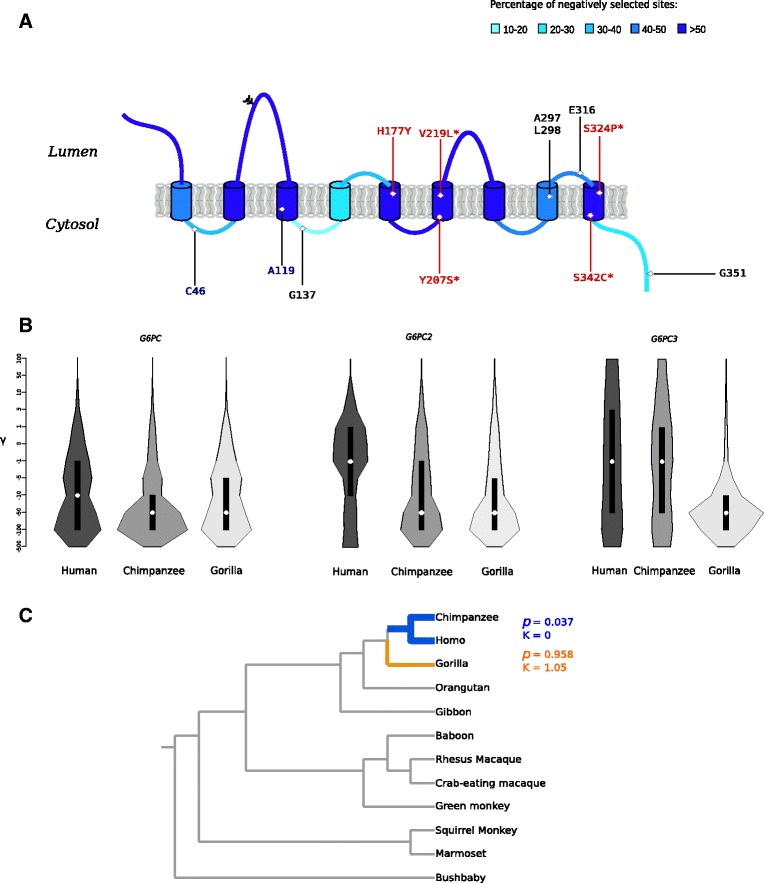
Evolutionary analysis of G6Pase genes. **a** G6PC2 is shown with its predicted membrane topology; protein regions are coloured in hues of blue according to the percentage of negatively selected sites (FEL *p* value < 0.1). Positively selected sites in the mammalian phylogeny (*black*) and in *Homininae* (*blue*) are reported on the structure. Missense variants associated with FG are shown in red. Asterisks denote negatively selected sites. The glycosylation site is also shown. **b** Violin plots of selection coefficients (median, white dot; interquartile range, black bar) for the three G6Pase genes. Selection coefficients (γ) are classified as strongly beneficial (100, 50), moderately beneficial (10, 5), weakly beneficial (1), neutral (0), weakly deleterious (−1), moderately deleterious (−5, −10), strongly deleterious (−50, −100), and inviable (−500). **c** Phylogenetic tree for primate *G6PC3* genes. Branches are color-coded according to RELAX results: blue, significant evidence of relaxed selection; orange, no significant evidence of relaxation

Positive selection may act on specific sites in a protein that is otherwise selectively constrained; to test for evidence of positive selection in the three G6Pase genes, we applied likelihood ratio tests (LRT) implemented in the *codeml* program [28, 29]. The total tree length for eutherian mammals sequences varied between 6.44 and 8.65 (Table 1); these values are within an optimal accuracy range for *codeml* sites models [30]. *codeml* was applied to compare models of gene evolution that allow (NSsite model M8 and M2a, positive selection models) or disallow (NSsite models M1a, M8a and M7, null models) a class of codons to evolve with dN/dS > 1. As reported in Table 2, all null models were rejected in favor of the positive selection models for *G6PC2*; the same result was obtained using different codon frequency models (F3x4 and F61) (Table 2). Conversely, no evidence of positive selection was obtained for *G6PC* and *G6PC3* (Additional file 1: Table S3). These results indicate that *G6PC2* alone evolved adaptively in mammals. The Bayes Empirical Bayes (BEB) analysis (from model M8) [30, 31] identified 5 codons showing strong evidence of positive selection (posterior probability > 0.95); most of these were also detected by FEL or REL (Table 2) [26]. With the exclusion of codon 137, selected sites were located in the C-terminal portion of the protein, often within highly constrained regions (Fig. 2a). Human coding polymorphisms that modulate glycemic traits are mainly located in this C-terminal highly constrained region (Fig. 2a); most of these variants affect codons that were targeted by negative selection during mammalian evolution (Fig. 2a).

**Table 2:** Likelihood ratio test statistics for models of variable selective pressure among sites in *G6PC2*

Codon frequency model	LRT Models	Degrees of freedom	−2ΔLnL^d^	*p* value	% of sites (average dN/dS)	Positively selected sites
F3x4						
	M1a vs M2a^a^	2	18.33	1.05x10^−4^	0.99% (2.72)	
	M7 vs M8^b^	2	46.36	8.58x10^−11^	4.67% (1.49)	G137 (BEB, REL, FEL), A297 (BEB), L298 (BEB, REL, FEL), E316 (BEB), G351 (BEB, REL)
	M8a^c^ vs M8	1	11.79	5.96x10^−4^		
F61						
	M1a vs M2a	2	9.15	1.03x10^−2^	0.75% (2.40)	
	M7 vs M8	2	39.46	2.69x10^−9^	4.93% (1.32)	
	M8a vs M8	1	6.68	9.77x10^−3^		

^a^M1a is a nearly neutral model that assumes one ω class between 0 and 1 and one class with ω = 1; M2a (positive selection model) is the same as M1a plus an extra class of ω > 1

^b^M7 is a null model that assumes that 0 < ω < 1 is beta distributed among sites; M8 (positive selection model) is the same as M7 but also includes an extra category of sites with ω > 1

^c^M8a is the same as M8, except that the 11th category cannot allow positive selection, but only neutral evolution

^d^2ΔlnL: twice the difference of the natural logs of the maximum likelihood of the models being compared

### Evolutionary analysis of G6Pase genes in humans and great apes

We next applied a population genetics-phylogenetics approach to study the evolution of G6Pase genes in the human, chimpanzee, and gorilla lineages. Specifically, we ran the gammaMap program [32] that jointly uses intra-specific variation and inter-specific diversity to estimate the distribution of fitness effects (i.e. population-scaled selection coefficients, γ) along coding regions. gammaMap categorizes codons into 12 classes of γ, ranging from strongly beneficial (γ = 100) to inviable (γ = −500); a γ equal to 0 indicates neutrality. The overall distribution of selection coefficients indicated that *G6PC* evolved under strong purifying selection in all lineages (median γ < −10, Fig. 2b). This was also the case for *G6PC2* in non-human primates (median γ = −100), whereas the human gene showed weaker constraint (Fig. 2b). Finally, the distribution of fitness effects for *G6PC3* was very different in distinct lineages. In fact, the codon distribution was almost homogeneous across the range of γ values in humans and chimpanzees, although the median remained below 0. In contrast, the gorilla lineage showed evidence of strong purifying selection (Fig. 2b). We thus assessed whether this pattern may derive from a relaxation of constraint in humans and chimpanzees. To test this possibility we applied the RELAX methodology [33] to the *G6PC3* primate phylogeny (Fig. 2c). Results were consistent with relaxed selection on the human/chimpanzee branches (*p* = 0.037, k = 0), but not on the gorilla lineage (*p* = 0.958, k = 1.05) (Fig. 2c). The same analysis for the human *G6PC2* branch revealed no relaxation of selective pressure (*p* = 0.866, k = 1.21). gammaMap also identified two positively selected codons (cumulative probability > 0.80 of γ ≥ 1) for human *G6PC2* (Fig. 2, Additional file 1: Table S4). One selected codon was also identified for human *G6PC3* (site 243), whereas no positively selected sites were detected for *G6PC* in any lineage.

### Evolutionary analysis in human populations

We finally investigated whether positive selection acted on G6Pase genes during the recent evolutionary history of human populations. Using the 1000 Genomes Phase 1 data for Yoruba, European, and Chinese we calculated pairwise F_ST_ [34], an estimate of population genetic differentiation. We also performed the DIND (Derived Intra-allelic Nucleotide Diversity) and iHS (integrated haplotype score) tests [35, 36] for all SNPs mapping to these genes. Statistical significance (in terms of percentile rank) for the F_ST_ statistic and for the DIND test was obtained by deriving empirical distributions. For the iHS test, absolute values higher than 2 were considered as significant [36]. No SNP in any G6Pase gene reached statistical significance (rank > 0.95) for both F_ST_ and for the DIND tests, and none had an |iHS| higher than 2. Overall, these results indicate that no variant/haplotype can be confidently called as positively selected. Likewise, nucleotide diversity (calculated as θ_W_ and π [37, 38]) for the entire gene regions was unexceptional if compared to those calculated for a reference set of 2000 genes. We conclude that G6Pase genes did not represent selection targets in recent human history.

### Association of *G6PC2* variants with T2D

**Fig. 3:**
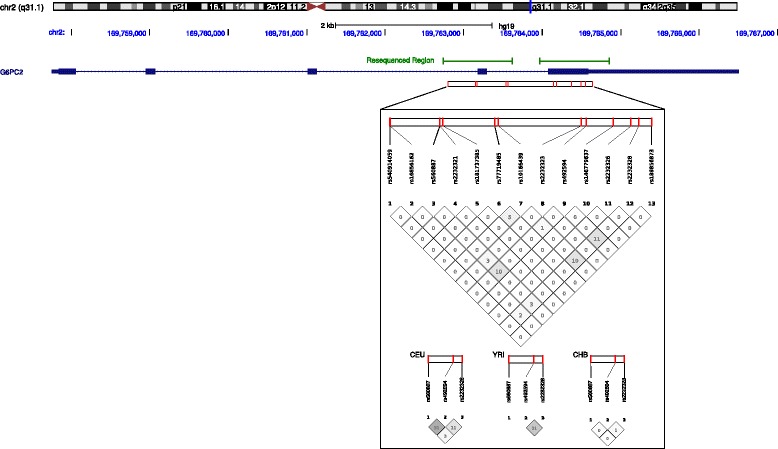
Linkage disequilibrium (LD) plots. The LD plot was constructed with Haploview 4.2 and displays *r* ^*2*^ values (× 100) for the polymorphic variants we identified. LD plots of the common variants for CEU, YRI and CHB is also shown. The exon-intron structure of *G6PC2* (*blue*) is also shown together with the two regions we resequenced (*green bars*)

Several genome-wide association studies (GWAS) have identified a functional non-coding variant (rs560887) in *G6PC2* that is associated with fasting glucose (FG) levels [3–7]. More recently, multiple rare and common coding variants in this gene were shown to influence FG [39, 40]. As mentioned above, all these coding variants are located in the two terminal exons of *G6PC2*, where most sites that are positively selected in mammals also map (Fig. 2a). The best characterized variants (H177Y, Y207S, V219L, and R283X) exert an effect independent of each other and of the GWAS SNP, indicating that haplotype analysis rather than single variant association is better suited to assess the contribution of *G6PC2* variants to metabolic traits [39, 40]. Despite their replicated effect on FG, the contribution of rare and common *G6PC2* variants to T2D susceptibility has remained controversial [6, 39, 41, 42]. We thus investigated a possible role for *G6PC2* variants in modulating the susceptibility to T2D in subjects from Saudi Arabia, a region with a high prevalence of metabolic disorders, including T2D [43, 44]. Specifically, we resequenced the two terminal exons of *G6PC2* (Fig. 3) in 562 subjects from Saudi Arabia, 185 of whom suffering from T2D (Additional file 1: Table S5). To limit phenotype heterogeneity only non-obese individuals (BMI < 30) were included. The rs560887 GWAS variant was also genotyped.

No novel missense or nonsense variant was detected in either T2D subjects or healthy controls (HC) and the frequency of known rare missense and nonsense variants was not significantly different in T2D and HC (Additional file 1: Table S6). Two common missense variants were nevertheless detected in the last *G6PC2* exon: rs492594 (V219L) and rs2232328 (S342C). The two variants display very limited linkage disequilibrium (LD) (Fig. 3). To address their contribution to T2D risk, logistic regression using age, sex, and BMI as covariates were used. After FDR correction for multiple tests, no association with T2D was observed (Table 3); conditioning on the GWAS variant, though, revealed a significant association for the two missense variants (Table 3). Haplotype analysis using the same covariates indicated above detected two haplotypes significantly associated with T2D (Table 4). Both the predisposing and the protective haplotype carry the glucose-raising allele at rs560887. The predisposing haplotype also includes the loss-of-function L219 allele (glucose-lowering) and the minor allele (C342) at rs2232328 (Table 4). These results should be regarded as preliminary due to the small sample size.

**Table 3:** Association of *G6PC2* variants with T2D

Sample/SNP (Variant)	Genotype frequency			Minor/Major allele	Minor allele freq (%)	Corrected *p* value	OR (IC 95%)	Corrected *p* value	OR (IC 95%)
						Unconditional		Conditional on rs560887	
rs560887, intronic, (GWAS)									
	CC	CT	TT						
Diabetic cohort	0.643	0.319	0.038	T/C	19.7	0.368	1.23 (0.78–1.95)	-	-
Control cohort	0.679	0.276	0.045		18.3				
rs492594 (p.Val219Leu)									
	GG	GC	CC						
Diabetic cohort	0.334	0.475	0.19	C/G	42.9	0.099	1.50 (1.03–2.18)	0.018	1.70 (1.14–2.54)
Control cohort	0.368	0.483	0.149		39.1				
rs2232328 (p.Ser342Cys)									
	CC	CG	GG						
Diabetic cohort	0.619	0.299	0.081	G/C	23.1	0.099	1.51 (0.97–2.36)	0.034	1.64 (1.04–2.58)
Control cohort	0.688	0.274	0.038		17.5

**Table 4:** *G6PC2* haplotype analysis

Haplotypes	Frequency in T2D (%)	Frequncy in unaffected (%)	OR	Association *p* value
rs560887 | rs492594| rs2232328				
CCG	20.16	13.27	2.00	0.007
CGC	34.00	40.94	0.62	0.017
CCC	25.20	25.31	1.02	0.916
TGC	18.56	16.52	1.31	0.280

Finally, to assess the effect of rare and common *G6PC2* variants on T2D risk, we applied a SNP-set based method, the Sequence Kernel Association Test (SKAT) [45]. SKAT was run either by inclusion of all variants identified through re-sequencing (*n =* 13, Fig. 3, Additional file 1: Tables S6 and S7) or by limiting analysis to missense SNPs plus the GWAS variant (rs560887). No significant association was detected. However, as for single-variant associations, the power of SKAT is limited when small samples are analyzed [45].

## Discussion

In this study we have analyzed the evolutionary history of three genes (*G6PC*, *G6PC2* and *G6PC3*) encoding the catalytic subunits of glucose-6-phosphatase, a central enzyme for glucose homeostasis. The analysis was motivated by the well-accepted concept that the availability of food resources is a driver of pivotal importance in the evolution in mammals and that, in natural settings, most mammals commonly face prolonged fasting and/or starvation [13]. Consequently, homeostatic mechanisms that sense plasma glucose levels and modulate them in response to the feeding status are expected to represent natural selection targets.

Commonly, positive and negative selection act in concert on the same protein-coding gene. In fact, due to structural and functional constraints, most amino acid replacements are deleterious and are eliminated by negative selection. Conversely, at a minority of sites, amino acid replacements may be favored because, without impairing protein function, they confer new advantageous properties [27]. In line with this view, we found all G6Pase genes to display an overall dN/dS lower than 1, indicating a preponderance of negative selection. Recent evidence showed that structural and folding requirements (i.e. the ability of a protein to fold properly and stably) represent major determinants of the evolutionary rate at protein sites [46]. The 3D structures of mammalian G6Pases has not been solved and we could not therefore assess whether among-site variation in evolutionary rates is correlated with parameters such as solvent accessibility or packing density [46]. Nonetheless, we reasoned that because the three proteins share considerable identity in terms of amino acid sequence and the same topological organization [1], they should also display a similar 3D structure and, consequently, corresponding residues should display similar evolutionary rates. In fact, this was only partially true, as the correlation of dN-dS at corresponding sites were either weak or non-significant. This suggests that, despite a similar membrane topology ad the maintenance of the catalytic function, mammalian G6Pases have evolved different structural features over time. Indeed, the three genes have been diverging for a long time, as the duplications that originated the three mammalian paralogs occurred during early vertebrate evolution. It is generally accepted [47] that two whole genome duplication events occurred in the lineage basal to all vertebrates, before the divergence of gnathostomes and cyclostomes, although some authors favored a model with a single whole genome duplication [48]. It is thus possible that *G6PC3* and the *G6PC/G6PC2* ancestor originated and were fixed after whole genome duplication(s) in the ancestral vertebrate. However, the basal position of one lamprey sequence with respect to gnathostome *G6PC* and *G6PC2* proteins suggests that the duplication event that originated the two genes occurred after the gnathostome/cyclostome split. After gene duplications, gene losses occurred in several species or lineages; for instance most marsupials and the platypus only have one *G6PC* gene. Additional *G6PC* duplications also occurred during vertebrate evolution; several bony fishes have 4 *G6PC* paralogs, possibly as a results of a whole genome duplication that occurred in the ancestor of teleosts [47]. A similar observation was reported for the rainbow trout, a glucose-intolerant fish, which displays 5 *G6PC* genes possibly fixed in this species after the salmonid-specific whole genome duplication [49]. Overall, these observations indicate that the *G6PC* gene family is highly dynamic and gene maintenance or loss in some lineages may be related to specific feeding needs or strategies.

In line with this view, we detected pervasive positive selection at mammalian *G6PC2* genes. Most residues targeted by selection are located in the C-terminal protein region, which is also subject to strong negative selection. Because of the role of G6PC2 as a glucose sensor, it is possible to speculate that adaptive changes in distinct mammals relate to trophic strategies including diet, hybernation, and feeding behavior. Interestingly, positively selected sites in the human *G6PC2* gene were detected as well. It is worth mentioning that the two selected residues are fixed or almost fixed in human populations; checking against the genome sequences of archaic hominins indicated that the C46 and A119 variant were already present in the genomes of Neandertals and Denisovans [50, 51]. These observations suggest that, as for other variants in metabolic genes [15], these changes were not driven to high frequency in humans as an adaptation to the dietary shift determined by agriculture. Indeed, population genetics analysis of modern human populations detected no recent selective event.

Unexpectedly, given its association with a human disease, two different analyses indicated that *G6PC3* genes have experienced a relaxation of selective pressure in the human and chimpanzee lineages. We note, however, that this finding does not imply that relaxed constraints are observed at all sites in the protein. Conversely, in humans this effect is driven by 4 nonsynonymous substitutions (either fixed or polymorphic relative to the common ancestor of Hominidae), including the positively selected 243 site, in the absence of synonymous substitution. Three of these changes are clustered in ~60 amino acid region (residues 216–275) suggesting that, for unknown reasons, this protein portion is tolerant to change in humans. To date, no SNC4 missense mutation has been described at these sites.

Among the three G6Pase genes, mutations in *G6PC2* have never been associated with a Mendelian human disease. This is in line with the mild phenotype of the KO mouse model, as well as with the observation that *G6PC2* is a pseudogene in rats. Moreover, recent functional data indicated that coding variants that reduce the expression of G6PC2, most likely by impairing its folding, segregate at appreciable frequency in human populations [39]. Notably, variants in *G6PC2* have been consistently associated with FG levels, whereas their contribution to T2D risk remains controversial. In particular, the rs560887 SNP is one of the strongest signals associated to FG (and related traits), and one of the most commonly replicated in large-scale analyses [3–6, 52–54]. Moreover, the variant was shown to be functional and to modulate *G6PC2* pre-mRNA splicing [7]. Although this latter finding does not necessarily imply that rs560887 is the causal variant, the effect of the glucose-raising allele (C) on increased splicing efficiency is suggestive [7]. However, distinct studies found either no association of rs560887 with T2D risk [42] or indicated a weak protective effect of the glucose-increasing allele [6, 41]. Recently, Mahajan and coworkers reported a glucose-increasing effect of the common V219 (rs492594-G) allele that modestly increases the risk of T2D as well [39]. The authors suggested that association analysis for *G6PC2* should be performed through haplotype reconstruction as multiple rare and common variants independently affect FG levels, and the direction of effect for rs492594 is reversed when analysis is conditioned on rs560887 [39]. Nonetheless, most large-scale analyses of T2D susceptibility performed single variant association tests, rather than haplotype inference, leaving the role of *G6PC2* in T2D partially unexplored.

Our sequencing analysis in the Saudi sample was motivated by the high prevalence of T2D in this population. The frequency of rare variants was not different in T2D and HC, but the small sample size is not well suited to this type of analysis. Haplotype analysis with common variants detected two haplotypes that associated with T2D susceptibility in Saudi subjects. The haplotypes include the rs2232328 (S342C) variant, that is not covered in exome chip arrays and was thus not analyzed in recent association studies of *G6PC2* variants for FG levels [39, 40]. In a genome-wide meta-analysis [53], rs2232328 showed a strong association with FG (*p* value adjusted for BMI = 5.1 × 10^−16^), which is likely independent of the lead variant rs560887, as their LD is low in all populations (r^2^ < 0.05) (http://analysistools.nci.nih.gov/LDlink/). The functional effect of the S342C substitution is presently unknown. Codon 342 is negatively selected in mammals and located in a highly constrained region; indeed, a cystein residue was present in all analyzed mammals with the only exception of macaques (Additional file 1: Figure S1). These observations suggest that the derived S342 allele impairs *G6PC2* function. Surprisingly, though, the V219 allele which also involves a negatively selected site and represents the ancestral state conserved in all mammals (with the only exception of the tree shrew), was recently shown to result in reduced function [39]. Indeed, G6PC2 molecules carrying the V219 allele are expressed at lower abundance due to proteasomal degradation [39]. This observation indicates that the functional effect of G6PC2 variants is difficult to predict, and in the case of the S342 substitution will need experimental testing.

The data we report herein, although preliminary, may help reconcile the contrasting results obtained for rs560887 on T2D risk, as its effect might depend on haplotype context and may vary in different populations depending on LD between rs560887 and other functional variants.

Clearly, further studies will be necessary to confirm the role of *G6PC2* variants on T2D susceptibility. First, the size of the Saudi sample is small and the associations we detected are weak, thus requiring validation in an independent larger sample. Second, variants in the 5′ region of *G6PC2* (rs13387347, rs1402837) and in the intergenic spacer downstream the transcription end site of the gene (rs563694) were also associated with FG [4, 55, 56]. These variants possibly contribute independently to FG levels and show variable levels of LD with the SNPs we analyzed. Because the focus of our work was on coding missense variants, we did not analyze these SNPs. However, they may contribute to T2D susceptibility either alone or in combinations with coding variants, warranting their inclusion in future efforts aimed at assessing the contribution of *G6PC2* genetic variability to T2D risk.

## Conclusions

In conclusion, we detected pervasive positive selection at mammalian *G6PC2* genes, with almost all selected sites located in the C-terminal portion of the protein.

We then investigated a possible role for *G6PC2* variants in modulating the susceptibility to T2D in subjects from Saudi Arabia. We detected two haplotypes, one predisposing and one protective, significantly associated with T2D. These preliminary results suggest that distinct *G6PC2* haplotypes modulate susceptibility to T2D.

## Methods

### Phylogenetic analysis in metazoans

Protein sequences of *G6PC* genes for 65 animal species were retrieved from the Ensembl Compara database (Additional file 1: Table S1). The genomes of the following metazoans were searched for *G6PC* orthologs and paralogs: *Strongylocentrotus purpuratus, Aplysia californica, Callorhinchus milii, Saccoglossus kowalevskii, Limulus polyphemus.* Searches were performed using BLASTp using the three human G6PC proteins as queries, as well as the two lamprey proteins and the single protein of sea urchin. All hits corresponded to predicted proteins derived from genomic sequences.

The genomes of three Cephalochordata (*Branchiostoma lanceolatum, Branchiostoma belcheri*, and *Asymmetron lucayanum*) was also searched for the presence of *G6PC* genes but no hit was obtained.

A maximum likelihood phylogenetic tree of 188 G6PC proteins was constructed using RAxML v8.2.9 [57] with 100 bootstrap replicates and the best protein substitution model automatically determinated by the software.

### Evolutionary analysis in mammals

Available mammalian sequences for *G6PC, G6PC2* and *G6PC3* were retrieved from the NCBI database (http://www.ncbi.nlm.nih.gov/). A list of species is available as Additional file 1: Table S2. DNA alignments were performed with the RevTrans 2.0 utility (http://www.cbs.dtu.dk/services/RevTrans/, MAFFT v6.240 as an aligner) [58], which uses the protein sequence alignment as a scaffold for constructing the corresponding DNA multiple alignment. All alignments were screened for the presence of recombination using GARD (Genetic Algorithm Recombination Detection) [25], a Genetic Algorithm implemented in the HYPHY suite [59]. Gene trees were generated by maximum-likelihood using phyML with a maximum-likelihood approach, a General Time Reversible (GTR) model plus gamma-distributed rates and 4 substitution rate categories [60].

The SLAC (Single-Likelihood Ancestor Counting) and FEL (Fixed Effects Likelihood) methods from the HYPHY package were used to calculate the overall dN/dS, to identify negatively selected sites (FEL significance cut-off = 0.1) and for calculating dN-dS (rate of nonsynonymous changes-rate of synonymous changes) at each site [26].

The site models implemented in PAML were developed to detect positive selection affecting only a few aminoacid residues in a protein. To detect selection, site models that allow (M2a, M8) or disallow (M1a, M7 and M8a) a class of sites to evolve with ω > 1 were fitted to the data using the F3x4 (codon frequencies estimated from the nucleotide frequencies in the data at each codon site) and the F61 (frequencies of each of the 61 non-stop codons estimated from the data) codon frequency model. Positively selected sites were identified using the Bayes Empirical Bayes (BEB) analysis (with a cut-off of 0.95). BEB calculates the posterior probability that each codon is from the site class of positive selection (under model M8) [30]. The REL (Random Effects Likelihood) [26] and FEL (with the default cutoff of 0.1) tools were also applied to identify positively selected sites. REL models variation in nonsynonymous and synonymous rates across sites according to a predefined distribution, with the selection pressure at an individual site inferred using an empirical Bayes approach; FEL directly estimates nonsynonymous and synonymous substitution rates at each site [26].

Tests for potential-relaxed selection of *G6PC2* and *G6PC3* genes in primates were performed using the hypothesis testing framework in RELAX from the HYPHY package [33]. RELAX calculates a selection intensity parameter, *k*, by taking into account that relaxation will exert different effects on sites subjected to purifying selection (*ω* < 1) and sites subjected to positive selection (*ω* > 1). Relaxation will move *ω* toward 1 for both categories. RELAX tests whether selection is relaxed or intensified on a subset of test branches compared with a subset of reference branches in a predefined tree. In the null model, the selection intensity is constrained to 1 for all branches, whereas in the alternative model *k* is allowed to differ between reference and test groups. The selection on test branches is intensified or relaxed compared with background branches when *k* > 1 or *k* < 1, respectively.

### Positive selection in Homininae

For gammaMap [32] analysis, genotype data from the phase 1 of the 1000 Genomes Project were retrieved from the dedicated website [61]; we retrieved SNP information for the three human populations: African (Yoruba), European, and Chinese. For the chimpanzee and gorilla analyses, genotype information were retrieved from [62] for 25 and 27 individuals, respectively.

Ancestral sequences were reconstructed by parsimony from the human, chimpanzee, orangutan and macaque sequences.

Analysis was performed assuming θ (neutral mutation rate per site), k (transitions/transversions ratio), and T (branch length) to vary among genes following log-normal distributions. For p (the probability that adjacent codons share the same population-scaled selection coefficient) we assumed a uniform distribution. For each gene we set the neutral frequencies of non-STOP codons (1/61). For selection coefficients, we considered a uniform Dirichlet distribution with the same prior weight for each selection class. For each gene we run 500,000 iterations with a 20,000 iteration burn-in and a thinning interval of 10 iterations.

### *G6PC* and *G6PC3* mutations

The list of G6PC and G6PC3 mutations was obtained from the Human Gene Mutation Database (HGMD, http://www.hgmd.cf.ac.uk/ac/) and the ClinVar database (http://www.ncbi.nlm.nih.gov/clinvar/). Only missense mutations were included in the analyses.

### Population genetics analyses

Genotype information from the Phase 1 of the 1000 Genomes Project were retrieved from the dedicated website (http://www.1000genomes.org/) [61].

Genotype information was obtained for the 3 genes (*G6PC*, *G6PC2* and *G6PC3*); in particular, three human populations with different ancestry were analyzed: Europeans (CEU), Africans (Yoruba,YRI), and East Asians (Han Chinese in Bejing, CHB). A control set of ~2,000 randomly selected genes was used as a reference set (hereafter referred to as control set). These gene were selected to be longer than 5000 bp and have more than 80% human-outgroup (chimpanzee, orangutan or macaque genomes) aligning bases; orthologous regions in the outgroups were retrieved using the LiftOver tool. These data were used to calculate θ_W_ [37], π [38] and their relative distruibutions.

The pairwise F_ST_ [34] and the DIND (Derived Intra-allelic Nucleotide Diversity) [35] test were calculated for all SNPs mapping to the analyzed genes, as well as for SNPs mapping to the control set. F_ST_ values are not independent from allele frequencies, so we binned variants in 50 classes based on the minor allele frequency (MAF) and calculated *F*_*ST*_ empirical distribution for each MAF class using the control set data. The same procedure was applied for the DIND test; thus, we calculated statistical significance by obtaining an empirical distribution of DIND values for variants located within control genes; in particular, the DIND test was calculated using a constant number of 40 flanking variants (20 upstream and 20 downstream), as previously described [63]. DIND values for the three human populations were binned in 100 derived allele frequency (DAF) classes, and for each class the distributions were calculated. As suggested [35], for values of iπ_D_ = 0 we set the DIND value to the maximum obtained over the corresponding class plus 20.

The iHS statistic was calculated as previously described [36] for all variants mapping to G6PC genes. Specifically, the iHS value was calculated using all SNPs surrounding each variant in a 5 kb region.

### Human subjects, genotyping and statistical analysis

A total of 562 subjects from the Biomarker Screening in Riyadh Project (RIYADH COHORT) were enrolled (Additional file 1: Table S5). Diagnosis of T2D was based on World Health Organization proposed cut-off: fasting plasma glucose > or = 7.0 mmol/L or 126 mg/dl. Subjects with medical complications (coronary artery disease, nephropathy, and end stage renal disease or liver disease) were excluded and a similar percentage of males and females was enrolled among T2D patients and controls. Anthropometry included measurement of height (to the nearest 0.5 cm) and weight (to the nearest 0.1 kg); BMI was calculated as kg/m^2^. According to the WHO criteria, individuals were classified as non obese if their BMI was < 30 kg/m^2^. Written consent was obtained from all participants, and ethical approval was granted by the Ethics Committee of the College of Science Research Center, King Saud University, Riyadh, Kingdom of Saudi Arabia (KSA).

The two terminal exons of *G6PC2* were resequenced through PCR amplification and direct sequencing (primer sequences are available upon request). PCR products were treated with ExoSAP-IT (USB Corporation Cleveland Ohio, USA), directly sequenced on both strands with a Big Dye Terminator sequencing Kit (v3.1 Thermo Fisher Scientific), and run on an Applied Biosystems ABI 3130 XL Genetic Analyzer. Sequences were assembled using DNA Baser Sequence Assembler version 4.10. A summary of all variants identified through resequencing is available (Fig. 3, Table 3, Additional file 1: Table S6 and S7).

Genetic association was investigated by logistic regression with age, sex and BMI as covariates, conditioning or not on the GWAS variant (rs560887). Analyses were performed using *PLINK* [64]*.*

The SKAT test is implemented in the SKAT R package [45]. The SKAT_commonRare function was used to combine the effect of common and rare variants. The suggested threshold (1/√2n, where n is the number of subjects) was used to define rare variants [45]. Analyses were performed either by deriving variant weights from a beta density function [45] or by using weights based on the minor allele frequency in the analyzed populations. As in PLINK analysis, age, sex and BMI were entered as covariates.

## Additional files

Additional file 1: Table S1. List of metazoan species used for the phylogenetic analysis. **Table S2.** List of mammalian species used for the evolutionary analysis. **Table S3.** Likelihood ratio test statistics for models of variable selective pressure among sites in G6PC and G6PC3. **Table S4.** Positively selected sites in G6PC2 and G6PC3 in the human, chimpanzee and gorilla lineages. **Table S5.** Characteristics of the Saudi cohort. **Table S6.** Rare missense variants in G6PC2. **Table S7.** Non-coding polymorphic variants in G6PC2. **Figure S1.** Multiple protein alignment of G6PC2 genes. (PDF 186 kb)

## Abbreviations

1000G: 1000 Genomes Pilot Project
BEB: Bayes Empirical Bayes
BMI: Body Mass Index
CEU: Europeans
CHB: Chinese
CHBJPT: (Chinese plus Japanese)
DAF: Derived Allele Frequency
DIND: Derived Intra-allelic Nucleotide Diversity
dN/dS: Non-synonymous substitution/synonymous substitution rate
FEL: Fixed Effects Likelihood
FG: Fasting Glucose
GARD: Genetic algorithm recombination detection
GWAS: Genome-Wide Association Study
LD: Linkage Disequilibrium
LRT: likelihood ratio test
MAF: Minor Allele Frequency
MEME: Mixed Effects Model of Evolution
PAML: Phylogenetic Analysis by Maximum Likelihood
REL: Random Effects Likelihood
SLAC: Single-likelihood ancestor counting
SNP: Single Nucleotide Polymorphism
T2D: Type 2 Diabetes
YRI: Yoruba
Znf: Zinc-finger
