# Supplementary material for: Susceptibility to type 2 diabetes may be modulated by haplotypes in G6PC2, a target of positive selection
Source: BMC Evol Biol. 2017 Feb 7;17:43. doi: 10.1186/s12862-017-0897-z (PMC5297017; doi:10.1186/s12862-017-0897-z)
Supplement: Additional file 1: Table S1. — List of metazoan species used for the phylogenetic analysis. Table S2. List of mammalian species used for the evolutionary analysis. Table S3. Likelihood ratio test statistics for models of variable selective pressure among sites in G6PC and G6PC3. Table S4. Positively selected sites in G6PC2 and G6PC3 in the human, chimpanzee and gorilla lineages. Table S5. Characteristics of the Saudi cohort. Table S6. Rare missense variants in G6PC2. Table S7. Non-coding polymorphic variants in G6PC2. Figure S1. Multiple protein alignment of G6PC2 genes. (PDF 186 kb) [file 12862_2017_897_MOESM1_ESM.pdf]

Table S1. List of metazoan species used for the phylogenetic analysis

**Common name**	***Scientific name***	**Class**	**Present in the Ensembl Compara database**
Alaskan stickleback	*Gasterosteus aculeatus*	Actinopterygii	Yes
Amazon molly	*Poecilia formosa*	Actinopterygii	Yes
Atlantic cod	*Gadus morhua*	Actinopterygii	Yes
Blind cave fish	*Astyanax mexicanus*	Actinopterygii	Yes
Common platy	*Xiphophorus maculatus*	Actinopterygii	Yes
Green spotted puffer	*Tetraodon nigroviridis*	Actinopterygii	Yes
Japanese medaka	*Oryzias latipes*	Actinopterygii	Yes
Nile tilapia	*Oreochromis niloticus*	Actinopterygii	Yes
Spotted gar	*Lepisosteus oculatus*	Actinopterygii	Yes
Tiger puffer	*Takifugu rubripes*	Actinopterygii	Yes
Zebra danio or zebrafish	*Danio rerio*	Actinopterygii	Yes
Western clawed frog	*Xenopus tropicalis*	Amphibia	Yes
Chicken	*Gallus gallus*	Aves	Yes
Collared flycatcher	*Ficedula albicollis*	Aves	Yes
Mallard	*Anas platyrhynchos*	Aves	Yes
Wild turkey	*Meleagris gallopavo*	Aves	Yes
Zebra finch	*Taeniopygia guttata*	Aves	Yes
Sea lamprey	*Petromyzon marinus*	Cephalaspidomorphi	Yes
elephant shark	*Callorhinchus milii*	Chondrichthyes	No
Sea urchin	*Strongylocentrotus purpuratus*	Echinoidea	No
acorn worms	*Saccoglossus kowalevskii*	Enteropneusta	No
sea hare	*Aplysia californica*	Gastropoda	No
Fruit fly	*Drosophila melanogaster*	Insecta	Yes
Algerian mouse	*Mus spretus*	Mammalia	Yes
Alpaca	*Vicugna pacos*	Mammalia	Yes
Armadillo	*Dasypus novemcinctus*	Mammalia	Yes
Bushbaby	*Otolemur garnettii*	Mammalia	Yes
Cat	*Felis catus*	Mammalia	Yes
Chimpanzee	*Pan troglodytes*	Mammalia	Yes
Cow	*Bos taurus*	Mammalia	Yes
Dog	*Canis familiaris*	Mammalia	Yes
Dolphin	*Tursiops truncatus*	Mammalia	Yes
Duck-billed platypus	*Ornithorhynchus anatinus*	Mammalia	Yes
Elephant	*Loxodonta africana*	Mammalia	Yes
Eurasian shrew	*Sorex araneus*	Mammalia	Yes
Ferret	*Mustela putorius furo*	Mammalia	Yes
Flying fox	*Pteropus vampyrus*	Mammalia	Yes
Gibbon	*Nomascus leucogenys*	Mammalia	Yes
Gorilla	*Gorilla gorilla*	Mammalia	Yes
Gray mouse lemur	*Microcebus murinus*	Mammalia	Yes
Green monkey	*Chlorocebus sabaeus*	Mammalia	Yes
Guinea pig	*Cavia porcellus*	Mammalia	Yes
Hoffmanns two-toed sloth	*Choloepus hoffmanni*	Mammalia	Yes
Horse	*Equus caballus*	Mammalia	Yes
House mouse	*Mus musculus*	Mammalia	Yes

Human	*Homo sapiens*	Mammalia	Yes
Little brown bat	*Myotis lucifugus*	Mammalia	Yes
Marmoset	*Callithrix jacchus*	Mammalia	Yes
Olive baboon	*Papio anubis*	Mammalia	Yes
Orangutan	*Pongo abelii*	Mammalia	Yes
Ords kangaroo rat	*Dipodomys ordii*	Mammalia	Yes
Panda	*Ailuropoda melanoleuca*	Mammalia	Yes
Philippine tarsier	*Tarsius syrichta*	Mammalia	Yes
Pika	*Ochotona princeps*	Mammalia	Yes
Rabbit	*Oryctolagus cuniculus*	Mammalia	Yes
Rhesus monkey	*Macaca mulatta*	Mammalia	Yes
Rock hyrax	*Procavia capensis*	Mammalia	Yes
Sheep	*Ovis aries*	Mammalia	Yes
Shorttailed opossum	*Monodelphis domestica*	Mammalia	Yes
Shrew	*Tupaia belangeri*	Mammalia	Yes
Squirrel	*Ictidomys tridecemlineatus*	Mammalia	Yes
Tammar wallaby	*Macropus eugenii*	Mammalia	Yes
Tasmanian devil	*Sarcophilus harrisii*	Mammalia	Yes
Tenrec	*Echinops telfairi*	Mammalia	Yes
Western European hedgehog	*Erinaceus europaeus*	Mammalia	Yes
Wild boar	*Sus scrofa*	Mammalia	Yes
Atlantic horseshoe crab	*Limulus polyphemus*	Merostomata	No
Chinese softshell turtle	*Pelodiscus sinensis*	Reptilia	Yes
Green anole	*Anolis carolinensis*	Reptilia	Yes
Coelacanth	*Latimeria chalumnae*	Sarcopterygii	Yes

Table S2. List of mammalian species used for the evolutionary analysis

**Common name**	***Scientific name***	***G6PC***	***G6PC2***	***G6PC3***
Aardvark	*Orycteropus afer*			
Alpaca	*Vicugna pacos*			
Armadillo	*Dasypus novemcinctus*			
Asinara white donkey	*Equus asinus*			
Baboon	*Papio hamadryas*			
Bactrian camel	*Camelus ferus*			
Big brown bat	*Eptesicus fuscus*			
Black flying-fox	*Pteropus alecto*			
Brush-tailed rat	*Octodon degus*			
Bushbaby	*Otolemur garnettii*			
Cape elephant shrew	*Elephantulus edwardii*			
Cape golden mole	*Chrysochloris asiatica*			
Cat	*Felis catus*			
Cheetah	*Acinonyx jubatus*			
Chimpanzee	*Pan troglodytes*			
Chinchilla	*Chinchilla lanigera*			
Chinese hamster	*Cricetulus griseus*			

Chinese tree shrew	*Tupaia chinensis*			
Cow	*Bos taurus*			
Crab-eating macaque	*Macaca fascicularis*			
Damaraland mole-rat	*Fukomys damarensis*			
Deer mouse	*Peromyscus maniculatus*			
Dog	*Canis lupus familiaris*			
Dolphin	*Tursiops truncatus*			
Domestic goat	*Capra hircus*			
Elephant	*Loxodonta africana*			
Ferret	*Mustela putorius furo*			
Flying fox	*Pteropus vampirus*			
Free-ranging baiji	*Lipotes vexillifer*			
Gibbon	*Nomascus leucogenys*			
Golden hamster	*Mesocricetus auratus*			
Gorilla	*Gorilla gorilla gorilla*			
Green monkey	*Chlorocebus sabaeus*			
Grey seal	*Halichoerus grypus*			
Guinea pig	*Cavia porcellus*			
Horse	*Equus caballus*			
Human	*Homo sapiens*			
Killer whale	*Orcinus orca*			
Lesser egyptian jerboa	*Jaculus jaculus*			
Little brown bat	*Myotis lucifugus*			
Manatee	*Trichechus manatus latirostris*			
Marmoset	*Callithrix jacchus*			
Minke whale	*Balaenoptera acutorostrata scammoni*			
Mouse	*Mus musculus*			
Naked mole-rat	*Heterocephalus glaber*			
Orangutan	*Pongo pygmaeus abelii*			
Pacific walrus	*Odobenus rosmarus divergens*			
Panda	*Ailuropoda melanoleuca*			
Pig	*Sus scrofa*			
Pika	*Ochotona princeps*			
Plains bison	*Bison bison bison*			
Polar bear	*Ursus maritimus*			
Rabbit	*Oryctolagus cuniculus*			
Rat	*Rattus norvegicus*			
Rhesus macaque	*Macaca mulatta*			
Sheep	*Ovis aries*			
Shrew	*Sorex araneus*			
Siberian tiger	*Panthera tigris altaica*			
Sperm whale	*Physeter catodon*			
Squirrel	*Spermophilus tridecemlineatus*			
Squirrel monkey	*Saimiri boliviensis*			
Star-nosed mole	*Condylura cristata*			
Sunda flying lemur	*Galeopterus variegatus*			
Tenrec	*Echinops telfairi*			
Tibetan antelope	*Pantholops hodgsonii*			

Water buffalo	*Bubalus bubalis*			
Weddell seal	*Leptonychotes weddelli*			
White rhinoceros	*Ceratotherium simum*			

Table S3. Likelihood ratio test statistics for models of variable selective pressure among sites in *G6PC* and *G6PC3*.

Gene/ LRTmodel	Degrees of freedom	-2ΔLnL[d]	*p* value	% of sites (average dN/dS)
G6PC				
M1a vs M2a[a]	2	0	1	6.33%(1)
M7 vs M8[b]	2	6.41	0.041	4.87% (1.04)
M8a[c] vs M8	1	2.93	0.087	
G6PC3				
M1a vs M2a	2	$2.00x10^{-6}$	0.999	2.78% (1)
M7 vs M8	2	4.07	0.131	3.52% (1.09)
M8a vs M8	1	1.69	0.194	

[a]M1a is a nearly neutral model that assumes one ω class between 0 and 1 and one class with ω=1; M2a (positive selection model) is the same as M1a plus an extra class of ω >1.
[b]M7 is a null model that assumes that 0<ω<1 is beta distributed among sites; M8 (positive selection model) is the same as M7 but also includes an extra category of sites with ω>1.
[c]M8a is the same as M8, except that the 11th category cannot allow positive selection, but only neutral evolution.
[d]2ΔlnL: twice the difference of the natural logs of the maximum likelihood of the models being compared.

Table S4. Positively selected sites in *G6PC2* and *G6PC3* in the human, chimpanzee and gorilla lineages.

Gene	Lineage	Codon	Ancestral AA	Derived AA	Pr[b]	MAF[c]
G6PC2						
	Human					
		46	Trp	Cys	0.808	0
		119	Ser	Ala	0.803	0.002
	Chimpanzee					
		ND[a]	ND	ND	ND	-
	Gorilla					
		ND	ND	ND	ND	-
G6PC3						
	Human					
		243	Met	Val	0.947	0
	Chimpanzee					
		ND	ND	ND	ND	-
	Gorilla					
		ND	ND	ND	ND	-

[a]ND:Non Detected

[b] Posterior probability of γ >0 as detected by gammaMap.
[c] Minor Allele Frequency

Table S5. Characteristics of the Saudi cohort

	T2D	Healthy controls
**Sample size**	185	377
**Females %**	48	49
**Males %**	52	51
**Age±s.d. (years)**	59.37 ± 11.93	37.05 ± 15.87
**BMI±s.d., kg/m2**	23.46 ± 3.20	21.88 ± 3.03

Table S6. Rare missense variants in *G6PC2*

SNP ID	Minor/Major allele	Substitution	Observed number (MAF[a])		*p* value	OR (IC 95%)	*p* value	OR (IC 95%)
			T2D	HC	Unconditional		Conditional on rs560887	
rs138726309	T/C	missense (H177Y)	0 (0)	0 (0)	NA	NA	NA	NA
rs2232323	C/A	missense (Y207S)	1 (0.53)	5 (1.33)	0.871	0.81 (0.06-11.00)	0.733	0.64 (0.05-8.57)
rs146779637	T/C	nonsense (R283X)	3 (1.64)	3 (0.80)	0.462	2.25 (0.26-19.41)	0.520	2.05 (0.23-18.13)
rs2232326	C/T	missense (S324P)	1 (0.55)	1 (0.27)	0.163	46.51 (0.21->100)	0.331	1.27 (0.78-2.06)

[a]MAF: minor allele frequency (%)

Table S7.Non-coding polymorphic variants in *G6PC2*

SNP/Sample	Genotype frequency (%)			Minor/Major allele	MAF[a]	*p* value Unconditional	*p* value Conditional on rs560887
**rs540914059 (intron)**							
	CC	CT	TT				
**Diabetic cohort**	1	0	0	T/C	0	0.999	0.999
**Control cohort**	0.997	0.003	0		0.132		
**rs16856162 (intron)**							
	GG	GA	AA				
**Diabetic cohort**	0.989	0.011	0	A/G	0.543	0.273	0.248
**Control cohort**	0.989	0.011	0		0.528		
**rs2232321 (intron)**							
	GG	GA	AA				
**Diabetic cohort**	0.984	0.016	0	A/G	0.806	0.299	0.214
**Control cohort**	0.992	0.008	0		0.396		
**rs181737385 (intron)**							
	GG	GA	AA				
**Diabetic cohort**	1	0	0	A/G	0	0.999	0.999
**Control cohort**	0.997	0.003	0		0.132		

**rs77719485 (intron)**							
	AA	AC	CC				
**Diabetic cohort**	1	0	0	C/A	0	0.999	0.999
**Control cohort**	0.997	0.003	0		0.132		
**rs10166439 (intron)**							
	CC	CT	TT				
**Diabetic cohort**	0.967	0.027	0.006	T/C	1.902	0.075[b]	0.089[c]
**Control cohort**	0.955	0.045	0		2.243		
**rs189856873 (3'-UTR)**							
	AA	AG	GG				
**Diabetic cohort**	1	0	0	G/A	0	0.999	0.999
**Control cohort**	0.997	0.003	0		0.135		

[a]MAF: minor allele frequency (%)
OR was calculated only for variants with MAF>1%
[b] OR= 0.176 (IC 95%: 0.03-1.19)
[c] OR= 0.191 (IC 95%: 0.03-1.29)

Figure S1. Multiple protein alignment of *G6PC2* genes. Positively selected sites are highlighted in yellow, sites identified by gammaMap are in blue, and the positions of human missense variants are shown in red.

```
                                                                   46
Human                  MDFLHRNGVLIIQHLQKDYRAYYTFLNFMSNVGDPRNIFFIYFPLCFQFNQTVGTKMIWV
Squirrel               MDFLHRNGVLIIQHLQKDYRAYYEFLNFMSNVGDPRNIFSIYFPLWFQLNQTVGTKMIWV
Mouse                  MDFLHRSGVLIIHHLQEDYRTYYGFLNFMSNVGDPRNIFSIYFPLWFQLNQNVGTKMIWV
Minke whale            MDFLHRNGVLIIQHLQKDYRAYYSFLNFMSNVGDPRNIFSIYFPLWFQLNQAVGTKMIWV
Chimpanzee             MDFLHRNGVLIIQHLQKDYRAYYTFLNFMSNVGDPRNIFFIYFPLWFQFNQTVGTKMIWV
Water buffalo          MDFLHRNGVLVIQHLQKDYRAYYNFLNFMSNVGDPRNIFSIYFPLWFQLNQTVGTKMIWV
Green monkey           MDFXHRNGVLIIQHLQKDYRAYYNFLNFMTNIGDPQNIFFIYFPLWFQLNQTVGTKMIWV
Polar bear             MDFLHRNGVLAIQHLQKDYRVYYNFLNFMSNVGDPRNIFSIYFPLWFPLNQTIGTKMIWV
Orangutan              MDFLHRNGVLIIQHLQKDYRAYYTFLNFMSNVGDPRNIFFIYFPLWFQLNQTVGTKMIWV
Marmoset               MDFLHRNEVLIIQQLQKDYQAYYNFLNFISNVGDPRNIFFIYFPLWFQLNQTVGTKMIRV
Sheep                  MDFLHRNGVLAIQHLQKDYRAYYSFLNFMSSVGDPRNIFAIYFPLWFQLNQTVGTKMIWV
Rabbit                 MDFLHRNGVLVIQHLQKDYRAYYDFLNFMSNVGDPRNIFSIYFPLWFQLNQTVGTKMIWV
Naked mole-rat         MDFLHRNGVLIIQHLQKDYRAYYDFLNFMSNVGDPRNIFSIYFPLWFQLNQTVGTKMIWV
Chinchilla             MDFLHRNGVLVIQHLQKDYRAYYDFLNFMSSVGDPRNIFSIYFPLWFQLNQTVGTKMIWV
Ferret                 MDFLHRNGVLVIQHLQKDYRAYYSFLNFMSNVGDPRNIFSIYFPLWFQLNQTVGTKMVWV
Elephant               MDFLHRNGVLVIQHLQKDYRAYYDFLNFMSSVGDPRNIFSIYFPLWFQLNQTVGTKMIWV
Capra                  MDFLHRNGVLAIQHLQKDYRAYYSFLNFMSSVGDPRNIFAIYFPLWFQLNQTVGTKMIWV
Pacific walrus         MDFLHRNGVLAIQHLQKDYRAYYNFLNFMSNVGDPRNIFSIYFPLWFQLNQTVGTKMIWV
Weddell seal           MDFLHRNGVLAIQHLQNDYRAYYNFLNFMSNVGDPRNIFSIYFPLWFQLNQTVGTKMIWV
Gibbon                 MDFLHRNGVLIIQHLQKDYRAYYTFLNFMSNVGDPRNIFSIYFPLWFQLNQTVGTKMIWV
```

```
Tenrec                 MDFLHRNGVLLIQHLQKDYRAYYDFLNFMSNAGDPRNIFSIYFPLWFQLNQTVGTKMIWV
Shrew                  MDFLHRDGVLIIQHLQKDYRAYYNFLNFMSNVGDPRNIFSIYFPLWFQLNQTVGTKMIWV
Horse                  MDFLHRNGVLIIQHLQKDYRAYYNFLNFMSSVGDPRNIFSIYFPLWFQLNQTVGTKMIWV
Guinea pig             MDFLHRNGVLVIQHLQKDYGAYYGFLNFMSSVGDPRNIFSIYFPLWFQLNQTVGTKMIWV
Brush-tailed rat       MDFLHKNGVLIIQHLQKDYRAYYDFLNFMSNVGDPRNIFSIYFPLWFQLNQTVGTKMIWV
Aardvark               MDFLHRNGVLVIQHLQKDYQGYYDFLNFMSNVGDPRNIFSIYFPLWFQLNQTVGTKMIWV
Dolphin                MDFFHRNGVLIIQHLQKDYRAYYSFLNFMSNVGDPRNIFSIYFPLWFQLNQAVGTKMIWV
Alpaca                 MDFLHRNGVLLIQHLQKDYRAYYNFLNFMSNVGDPRNIFSIYFPLWFQLNQTVGTKMIWV
Manatee                MDFLHRNGVLVIQHLQRDYRAYYDFLNFMSNVGDPRNIFSIYFPLWFQLNQTVGTKMIWV
Tree shrew             MDFLHRSGVLIIQHLQNDYRTYYNFLNFMSNVGDPRNSFSIYFPLWFQLNHTVGTKMIWV
Baboon                 MDFPYMNGVLIIQHLQKDYRAYYNFLNFMSNIGDPQNIFFIYFPLWFQLNQTVGTKMIWV
Rhesus macaque         MDFXHRNGVLIIQHLQKDYRAYYNFLNFMSNIGDPQNIFFIYFPLWFQLNQTVGTKMIWV
Cheetah                MDFLHRNGVLVIQHLQKDYRAYYNFLNFMSNVGDPRNIFSIYFPLWFQLNQTVGTKMIWV
Big brown bat          MDFLHRNGVLIIQHLQKDYRAYYNFLNFMSNVGDPRNIFSIYFPLWFQLNQTVGTKMIWV
Sperm whale            MDFLHRNGVLIIQHLQKDYRAYYSFLNFMSNVGDPRNIFSIYFPLWFQLNQAVGTKMIWV
Little brown bat       MDFLHRNGVLIIQHLQKDYRAYYNFLNFMSNVGDPRNIFSIYFPLWFQLNQTVGTKMIWV
Cow                    MDFLHRNGVLVIQHLQKDYRAYYNFLNFMSNVGDPRNIFSIYFPLWFQLNQTVGTKMIWV
Armadillo              MDFLHRNGVLVIQHLQKDYRAYYDFLNFMSNVGDPRNIFSIYFPLWFQLNQTVGTKMIWV
Sunda flying lemur     MDFLHRNGVLIIQHLQKDYRAYYDFLNFMSNVGDPRNIFSIYFPLWFQLNQTVGTKMIWV
Star-nosed mole        MDFLHRNGVLIIQHLQKDYRTYYNFLNFMSNVGDPRNIFSIYFPLWFQLNQTVGTKMIWV
Bactrian camel         MDFLHRNGVLLIQHLQKDYRAYYSFLNFMSNVGDPRNIFSIYFPLWFQLNQTVGTKMIWV
Gorilla                MDFLHRNGVLIIQHLQKDYRAYYTFLNFMSNVGDPRNIFFIYFPLWFQLNQTVGTKMIWV
Pig                    MDFLHRNGVLLIQHLQKDYRAYYSFLNFMSNVGDPRNIFSIYFPLWFQLNQRVGTKMIWV
Plains bison           MDFLHRNGVLVIQHLQKDYRAYYNFLNFMSNVGDPRNIFSIYFPLWFQLNQTVGTKMIWV
Flying fox             MDFLHRNGVLIIQHLQKDYRAYYNFLNFMSNVGDPRNIFSIYFPLWFQLNQTVGTKMIWV
Golden hamster         MDFFHSSGVLIIQHLQKDYRAYYDFLNFMSNVGDPRNVFSIYFPLWFQLNQTVGTKMIWV
Cape golden mole       MDFLHRNGILFIQHLQKDYRAYYDFLNFMSNVGDPRNIFSIYFPLWFQLNQTVGTKMIWV
Cape elephant shrew    MDFLHRNGVLVIQHLQKDYRAYYDFLNFMSNVGDPRNIFSIYFPLWFQLNQTVGTKMIWV
Squirrel monkey        MDFLHRNGVLIIQHLQKDYRAYYNFLNFMSNVGDPRNIFFIYFPLWFQLNQTVGTKMIWV
Cat                    MDFLHRNGVLVIQHLQKDYRAYYNFLNFMSNVGDPRNIFSIYFPLWFQLNQTVGTKMIWV
Bushbaby               MDFLHRNGVLIIQNLQKDYRACYNFLNFMSKVGEPRNIFSIYFPLWFQLNQTVGTKMIWV
Killer whale           MDFLHRNGVLIIQRLQKDYRAYYSFLNFMSNVGDPRNIFSIYFPLWFQLNQAVGTKMIWV
Pika                   MDFLHWNGVLIIQHLQKDYRAYYDFLNFMSNVGDPRNIFTIYFPLWFQLNQTVGTKMIWV
Tiger                  MDFLHRNGVLVIQHLQKDYRAYYNFLNFMSNVGDPRNIFSIYFPLWFQLNQTVGTKMIWV
Black flying-fox       MDFLHRNGVLIIQHLQKDYRAYYNFLNFMSNVGDPRNIFSIYFPLWFQLNQTVGTKMIWV
Panda                  MDFLHRNGVLAIQHLQKDYRAYYNFLNFMSNVGDPRNIFSIYFPLWFQLNQTIGTKMIWV
Tibetan antelope       MDFLHRNGVLVIQHLQKDYRAYYNFLNFMSNVGDPRNIFSIYFPLWFQLNQTVGTKMIWV
Free-ranging baiji     MDFLHRNGVLVIQHLQKDYRAYYSFLNFMSNVGDPRNIFSIYFPLWFQLNQAVGTKMIWV
Lesser jerboa          MDFLHRNGVLIIQHLQKDYRAYYDFLNFMSSVGDPRNIFSIYFPLWFQLNQTVGTKMIWV
Dog                    MDFLHRNGVLAIQHLQKDYRAYYNFLNFMSNVGDPRNIFSIYFPLWFQLNQTIGTKMIWV
White rhinoceros       MDFLHRNGVLVIQHLQKDYRAYYNFLNFMSSVGDPRNIFSIYFPLWFQVNQRVGTKMIWV
White donkey           MDFLHRNGVLIIQHLQKDYRAYYNFLNFMSSVGDPRNIFSIYFPLWFQLNQTVGTKMIWV
Crab-eating macaque    MDFXHRNGVLIIQHLQKDYRAYYNFLNFMSNIGDPQNIFFIYFPLWFQLNQTVGTKMIWV
Chinese hamster        MDFLHRSRVLIIQHLQKDYRAYYNFLNFMSNVGDLRNIFSIYFPLWFQLNQTVGTKMIWV

                                                                                 119
Human                  AVIGDWLNLIFKWILFGHRPYWWVQETQIYPNHSSPCLEQFPTTCETGPGSPSGHAMGAS
Squirrel               AVIGDWFNLIFKWILFGHRPYWWIQETQIHPNHSSACLEQFPTTCETGPGSPSGHAMGSS
Mouse                  AVIGDWFNLIFKWILFGHRPYWWIQETEIYPNHSSPCLEQFPTTCETGPGSPSGHAMGSS
Minke whale            AVVGDWFNLIFKWILFGHRPYWWVQETQIYPNHSSPCLEQFPTTCETGPGSPSGHAMGSS
Chimpanzee             AVIGDWLNLIFKWILFGHRPYWWVQETQIYPNHSSPCLEQFPTTCETGPGSPSGHAMGSS
Water buffalo          AVIGDWFNLIFKWILFGHRPYWWVQETQIYPNHSSPCLEQFPTTCETGPGSPSGHAMGSS
Green monkey           AVIGDWFNLIFKWILFGHRPYWWVQETQIYPNHSSPCLEQFPTTCETGPGSPSGHAMGSS
Polar bear             AVIGDWFNLIFKWILFGHRPYWWVQETQIYPNHSSPCLEQFPTTCETGPGSPSGHAMGSS
Orangutan              AVIGDWFNLIFKWILFGDRPYWWVQETQIYPNHSSPCLEQFPTTCETGPGSPSGHAMGSS
Marmoset               AVIGDWFNLIFKWILFGHRPYWWVQETQIYPNHSSPCLEQFPTTCETGPGSPTGHAMGLS
Sheep                  AVIGDWFNLIFKWILFGHRPYWWVQETQIYPNHSSPCLEQFPTTCETGPGSPSGHAMGSS
Rabbit                 AVIGDWFNLIFKWILFGHRPYWWVQETQIYPNHSSPCLEQFPTTCETGPGSPSGHAMGSS
Naked mole-rat         AVIGDWFNLIFKWVLFGHRPYWWVQETQIYQNHSSSCLEQFPTTCETGPGSPSGHAMGSS
Chinchilla             AVIGDWFNLIFKWILFGHRPYWWVQETQIYPNHSSLCLEQFPTTCETGPGSPSGHAMGSS
Ferret                 AVIGDWFNLIFKWILFGHRPYWWVQETQIYANHSSPCLEQFPTTCETGPGSPSGHAMGSS
Elephant               AVIGDWFNLIFKWILFGHRPYWWVQETQIYPNHSSPCLEQFPTTCETGPGSPSGHAMGSS
Capra                  AVIGDWFNLIFKWILFGHRPYWWVQETQIYPNHSSPCLEQFPTTCETGPGSPSGHAMGSS
Pacific walrus         AVIGDWFNLIFKWILFGHRPYWWVQETQIYPNHSSPCLEQFPTTCETGPGSPSGHAMGSS
Weddell seal           AVIGDWFNLIFKWILFGHRPYWWVQETQIYPNHSSPCLEQFPTTCETGPGSPSGHAMGSS
Gibbon                 AVIGDWFNLIFKWILFGHRPYWWVQETQIYPNHSSPCLEQFPTTCETGPGSPSGHAMGSS
Tenrec                 AVIGDWFNLIFKWILFGHRPYWWVHETQIYPNHSSLCLEQFRATCETGPGSLXGHAMGSS
```

```
Shrew                  AVIGDWFNLIFKWILFGHRPYWWVQETQIYPNYSSPCLEQFPATCETGPGSPSGHAMGSF
Horse                  AVVGDWFNLIFKWILFGHRPYWWVQETQIYPNHSSPCLEQFPTTCETGPGSPSGHAMGSS
Guinea pig             AVIGDWFNLIFKWILFGHRPYWWVQETQIYPNHSSLCLEQFPTTCETGPGSPSGHAMGSS
Brush-tailed rat       AVIGDWFNLIFKWILFGHRPYWWVQETQLYPNHSNLCLEQFPTTCETGPGSPSGHAMGSS
Aardvark               AVIGDWFNLIFKWILFGHRPYWWVQETQIYPNHSSPCLEQFPTTCETGPGSPSGHAMGSS
Dolphin                AVVGDWFNLIFKWILFGHRPYWWVQETQIYPNHSSPCLEQFPTTCETGPGSPSGHAMGSS
Alpaca                 AVIGDWFNLIFKWILFGHRPYWWVQETQIYPNHSNPCLEQFPTTCETGPGSPSGHAMGSS
Manatee                AVIGDWFNLIFKWILFGHRPYWWVQETQIYPNHSSPCLEQFPTTCETGPGSPSGHAMGSS
Tree shrew             AVIGDWFNLIFKWILFGHRPYWWIQETHIYPNHSSPCLEQFPTTCETGPGSPSGHAMGSS
Baboon                 AVTGDWFNLIFKWILFGHRPYWWVQETQIYPNHSSPCLEQFPTTCETGPGSPSGHAMGSS
Rhesus macaque         AVIGDWFNLIFKWILFGHRPYWWVQETQIYPNHSSPCLEQFPTTCETGPGSPSGHAMGSS
Cheetah                AVIGDWFNLIFKWILFGHRPYWWVQETQIYPNHSSSCLEQFPTTCETGPGSPSGHAMGSS
Big brown bat          AVIGDWFNLIFKWILFGHRPYWWIQETQIYLNRSGPCLEQFPTTCETGPGSPSGHAMGSS
Sperm whale            AVVGDWFNLIFKWILFGHRPYWWVQETQIYPNHSSPCLEQFPTTCETGPGSPSGHAMGSS
Little brown bat       AVIGDWFNLIFKWILFGHRPYWWIQETQIYPNRSSPCLEQFPTTCETGPGSPSGHAMGSS
Cow                    AVIGDWFNLIFKWILFGHRPYWWVQETQIYPNHSSPCLEQFPTTCETGPGSPSGHAMGSS
Armadillo              AVMGDWFNLIFKWILFGHRPYWWIQETQIYPNHSSPCLEQFPTTCETGPGSPSGHAMGSA
Sunda flying lemur     AVVGDWFNLIFKWILFGHRPYWWVQETQIYPNHSSPCLEQFPTTCETGPGSPSGHAMGSS
Star-nosed mole        AVIGDWFNLIFKWILFGHRPYWWVQENQIYSNHSSPCLEQFPTTCETGPGSPSGHAMGSS
Bactrian camel         AVIGDWFNLIFKWILFGHRPYWWVQETQIYPNHSNPCLEQFPTTCETGPGSPSGHAMGSS
Gorilla                AVIGDWLNLIFKWILFGHRPYWWVQETQIYPNHSSPCLEQFPTTCETGPGSPSGHAMGSS
Pig                    AVIGDWFNLIFKWILFGHRPYWWVQETQIYPNHSSPCLEQFPTTCETGPGSPSGHAMGSS
Plains bison           AVIGDWFNLIFKWILFGHRPYWWVQETQIYPNHSSPCLEQFPTTCETGPGSPSGHAMGSS
Flying fox             AVIGDWFNLIFKWILFGHRPYWWVQETQIYPNHSSPCLEQFPTTCETGPGSPSGHAMGSS
Golden hamster         AVIGDWFNLIFKWILFGHRPYWWIQETQVYPNHSSPCLEQFPTTCETGPGSPSGHAMGSL
Cape golden mole       AVIGDWFNLICKWILFGHRPYWWVQETQNYPNHSRLCLEQFPTTCETGPGSPSGHAMGSS
Cape elephant shrew    AVIGDWFNLIFKWILFGHRPYWWVQETQIYPNHSSPCLEQFPTTCETGPGSPSGHAMGSS
Squirrel monkey        AVIGDWFNLIFKWILFGHRPYWWVQETQIYPNHSSPCLEQFPTTCETGPGSPSGHAMGSS
Cat                    AVIGDWFNLIFKWILFGHRPYWWVQETQIYPNHSSSCLEQFPTTCETGPGSPSGHAMGSS
Bushbaby               AVIGDWFNLILKWILFGHRPYWWVQETQIYPNHSSPCLEQFPTTCETGPGSPSGHAMGSS
Killer whale           AVVGDWFNLIFKWILFGHRPYWWVQETQIYPNHSSLCLEQFPTTCETGPGSPSGHAMGSS
Pika                   AVIGDWFNLIFKWILFGHRPYWWVQETQIYTNHSSPCLEQFPTTCETGPGSPSGHAMGSS
Tiger                  AVIGDWFNLIFKWILFGHRPYWWVQETQIYPNHSSSCLEQFPTTCETGPGSPSGHAMGSS
Black flying-fox       AVIGDWFNLIFKWILFGHRPYWWVQETQIYPNHSSPCLEQFPTTCETGPGSPSGHAMGSS
Panda                  AVIGDWFNLIFKWLLFGHRPYWWVQETQIYPNHSSPCLEQFPTTCETGPGSPSGHAMGSS
Tibetan antelope       AVIGDWFNLIFKWILFGHRPYWWVQETQIYPNHSSPCLEQFPTTCETGPGSPSGHAMGSS
Free-ranging baiji     AVVGDWFNLIFKWILFGHRPYWWVQETQIYPNHSSPCLEQFPTTCETGPGSPSGHAMGSS
Lesser jerboa          AVIGDWFNLIFKWILFGHRPYWWIQETQIYPNHSSPCLEQFPTTCETGPGSPSGHAMGSS
Dog                    AVIGDWFNLIFKWILFGHRPYWWVQETQIYPNHSSPCLEQFPTTCETGPGSPSGHAMGSS
White rhinoceros       AVIGDWFNLIFKWILFGHRPYWWVQETQIYPNHSNPCLEQFPTTCETGPGSPSGHAMGSS
White donkey           AVVGDWFNLIFKWILFGHRPYWWVQETQIYPNHSSPCLEQFPTTCETGPGSPSGHAMGSS
Crab-eating macaque    AVIGDWFNLIFKWILFGHRPYWWVQETQIYPNHSSPCLEQFPTTRETGPGSPSGHAMGSS
Chinese hamster        AVIGDWFNLIFKWILFGHRPYWWIQETQIYPNHSSPCLEQFPTTCETGPGSPSGHAMGSS

                                      137                                     177
Human                  CVWYVMVTAALSHTVCGMDKFSITLHRLTWSFLWSVFWLIQISVCISRVFIATHFPHQVI
Squirrel               CVWYVMVTAALSHAVSRMDEFSTTLHRLTWSFLWSLFWLIQISVCISRVFIATHFPHQVI
Mouse                  CVWYVMVTAALSYTISRMEESSVTLHRLTWSFLWSVFWLIQISVCISRVFIATHFPHQVI
Minke whale            CVWYVMVTAALGHTISRMDKSFTTLHRLTWSFLWSLFWLIQISVCISRVFIATHFPHQVI
Chimpanzee             CVWYVMVTAALSHTVCGMDKFSITLHRLTWSFLWSVFWLIQISVCISRVFIATHFPHQVI
Water buffalo          CVWYVMVTEALSHAISQMDKSFITLHRLTWSFLWSLFWLIQISVCISRVFIATHFPHQVI
Green monkey           CVWYVMVTAALSHTVCGMDKFFITLHRLTWSFLWSVFWLIQISVCISRVFIATHFPHQVI
Polar bear             CVWYVMVTAALSHTVSRMDKSLTILHRLTWSFLWSLFWLIQISVCISRVFIATHFPHQVI
Orangutan              CVWYVMVTAALSHTVCGMDKFSITLHRLTWSFLWSVFWLIQISVCISRVFIATHFPHQVI
Marmoset               CVWYVMIPAALSHTVCRMDKVSITLHRLTWSFLWSVFWLIQISVCIFRVFIATHFPHQVI
Sheep                  CVWYVMVTAALSHTVSQMDRSFITLHRLTWSFLWSLFWLIQISVCISRVFIATHFPHQVI
Rabbit                 CVWYVMVTAALSHTVSRMDKFSVTLHRLTWSFLWSLFWLIQISVCISRVFIATHFPHQVI
Naked mole-rat         CVWYVMVTAALSRSVSPMDKFSVTRHRLTWSFLWSLFWLIQISVCISRVFIATHFPHQVI
Chinchilla             CVWYVMVTAALSHSVGRMDKFSITLHRLTWSFLWSLFWLIQISVCISRVFIATHFPHQVI
Ferret                 CVWYVMITAALGHTVGRMDKSLTMLYRLTWSFLWSLFWLIQISVCISRVFIATHFPHQVI
Elephant               CVWYVMVTAALSYTVSQLDKSSITFHRLTWSFLWSLFWLIQISVCISRVFIATHFPHQVI
Capra                  CVWYVMVTAALSHTVSQMDKSFITLHRLTWSFLWSLFWLIQISVCISRVFIATHFPHQVI
Pacific walrus         CVWYVMVTAALSHTVSQMDKSLTTLHRLTWSFLWSLFWLIQISVCISRVFIATHFPHQVI
Weddell seal           CVWYVMVTAALSRTVSRMDKSLTTLHRLTWSFLWSLFWLIQISVCISRVFIATHFPHQVI
Gibbon                 CVWYVMVTAALSHTDCGMDKFSITLHRLTWSFLWSVFWLIQISVCISRVFIATHFPHQVI
Tenrec                 CVWYVMVTAALSDAVGRTDKSSLILHRLTWSFLWSLFWVIQISVCISRVFIATHFPHQVI
Shrew                  CVWYVMVTAALSPSVCRIEKWSTTLHRLTRSLLWSLFWLIQISVCISRVFIATHFPHQVI
```

```
Horse                  CVWYVMVTAALSHTVSQGDKSSTTLHRLTWSFLWSLFWLIQISVCISRVFIATHFPHQVI
Guinea pig             CVWYVMVTAALSHSVGRMDKFSITLHRLTWSFLWSLFWLIQISVCISRVFIATHFPHQVI
Brush-tailed rat       CVWYVMVTAALSHSVGRMDKFSVTLHRLTWSFLWSLFWLIQISVCISRVFIATHFPHQVI
Aardvark               CVWYVMVTAALSYSVTWIDKSSITLHRLTWSFLWSLFWLIQISVCISRVFIATHFPHQVI
Dolphin                CVWYVMVTAALGHTISRMDKSFPTLHRLTWSFLWSLFWLIQISVCISRVFIATHFPHQVI
Alpaca                 CVWYVMVTAALGHMVNQMDKLSNTLHRLTWSFLWSLFWLIQISVCISRVFIATHFPHQVI
Manatee                CVWYVMVTAALSYTVSRLDKSSITLHRLTWSFLWSLFWLIQISVCISRVFIATHFPHQVI
Tree shrew             CVWYVMVTAALSHTVSWLDKFSITLHRLTWSFLWSLFWLIQISVCISRVFIATHFPHQVI
Baboon                 CVWYVMVTAALSHTVCGMDKFSITPHRLTWSFLWSVFWLIPISVCISRVFIATHFPHQVI
Rhesus macaque         CVWYVMVTAALSHTVCGMDKFSITLHRLTWSFLWSVFWLIQISVCISRVFIATHFPHQVI
Cheetah                CVWYVMVTAALSHTVSRMDKSSTTLHRLTWSFLWSLFWLIQISVCISRVFIATHFPHQAI
Big brown bat          CVWYVMVTAALSHTVSRMDESSTTLHRLTWSFLWSLFWLIQISVCISRVFIATHFPHQVI
Sperm whale            CVWYVMVTAALGHTISWMDKSFTTLHRLTWSFLWSLFWLIQISVCISRVFIATHFPHQVI
Little brown bat       CVWYVMVTAALSNTVSWMDKSSTTLHRLTWSFFWSIFWLIQISVCISRVFIATHFPHQVI
Cow                    CVWYVMVTAALSHTVSQMDKSFITLHRLTWSFLWSLFWLIQINVCISRVFIATHFPHQVI
Armadillo              CVWYVMVTAALSYTVPQKDESSITLHRLTWSFLWSLFWLIQISVCISRIFIATHFPHQVI
Sunda flying lemur     CVWYVMVTAVLSHTVSQMDKFSITLHRLTWSFLWSLFWLIQISVCISRVFIATHFPHQVI
Star-nosed mole        CVWYIMITATLSHTVSQMDKLSTTLHRLTWSFLWSLFWLIQISVCISRVFIATHFPHQVI
Bactrian camel         CVWYVMVTAALGHMVNQMDKLSTTLHRLTWSFLWSLFWLIQISVCISRVFIATHFPHQVI
Gorilla                CVWYVMVTAALSHTVCGMDKFSITLHRLTWSFLWSVFWLIQISVCISRVFIATHFPHQVI
Pig                    CVWYVMVTAALGHTIRWMDKSSTTLHRLTWSFLWSLFWLIQISVCISRVFIATHFPHQVI
Plains bison           CVWYVMVTAALSHTDSQMDKSFITLHRLTWSFLWSLFWLIQISVCISRVFIATHFPHQVI
Flying fox             CVWYVMITAALSHTVSRMDKSSITLHRLTWSFLWSLFWLIQISVCISRVFIATHFPHQVI
Golden hamster         CVWYVMVTAALSYTVSSLDKSFVTLHRLTWSFLWSVFWLIQVSVCISRVFIATHFPHQVI
Cape golden mole       CVWYVMVTAALSYAVSRIDKSSITLHRLTWSFLWSLFWVIQFTVCISRVFIATHFPHQVI
Cape elephant shrew    CVWYVMVTAALSYTVNHTDKSSITLHRLTWSFLWSLFWLIQISVCISRVFIATHFPHQVI
Squirrel monkey        CVWYVMITAALSHTVCRMDKVSITLHRLTWSFLWSVFWLIQISVCISRVFIATHFPHQVI
Cat                    CVWYVMVTAALSHSVSRMDKSSTTLHRLTWSFLWSLFWLIQISVCISRVFIATHFPHQAI
Bushbaby               CVWYVMVTAALSHTVTPVEKFSITLHRLTWSCLWSLYWLIQISVCISRVFIATHFPHQVI
Killer whale           CVWYVMVTAALGHTISRMDKSFTTLHRLTWSFLWSLFWLIQISVCISRVFIATHFPHQVI
Pika                   CVWYVMVTAALSHTVSRMDKFSITLHRLTWSFLWSLFWLIQISVCISRVFIATHFPHQVI
Tiger                  CVWYVMVTAALSHTVSRMDKSSTTLHRLTWSFLWSLFWLIQISVCISRVFIATHFPHQAI
Black flying-fox       CVWYVMITAALSHTVSRMDKSSITLHRLTWSFLWSLFWLIQISVCISRVFIATHFPHQVI
Panda                  CVWYVMVTAALSHTVSRMDKSLTILHRLTWSFLWSLFWLIQISVCISRVFIATHFPHQVI
Tibetan antelope       CVWYVMVTAALSHTVSQMDKSFIILHRLTWSFLWSLFWLIQISVCISRVFIATHFPHQVI
Free-ranging baiji     CVWYVMVTAALGHTISRMDKSFTTLHRLTWSFLWSLFWLIQISVCISRVFIATHFPHQVI
Lesser jerboa          CVWYVMVTAALSHTVSWMDKFSVTLHRLTWSCLWSLFWLIQISVCISRVFIATHFPHQVI
Dog                    CVWYVMVTAALSHTVSRMDKSLTTLHRLTWSFLWSLFWLIQISVCISRVFIATHFPHQVI
White rhinoceros       CVWYVMVTAALSHTVSQRDKSSTTLHRLTWSFLWSLFWLIQISVCISRVFIATHFPHQVI
White donkey           CVWYVMVTAALSHTVSQGDKSSTTLHRLTWSFLWSLFWLIQISVCISRVFIATHFPHQVI
Crab-eating macaque    CVWYVMVTAALSHTVCGMDKFSITLHRLTWSFLWSVFWLIQISVCISRVFIATHFPHQVI
Chinese hamster        CVWYVMVTAALSYTVSRLDKSSVTLHRLTWSCLWSVFWLIQVSVCISRVFIATHFPHQVI


                                                207         219
Human                  LGVIGGMLVAEAFEHTPGIQTASLGTYLKTNLFLFLFALGFYLLLRVLNIDLLWSVPIAK
Squirrel               LGVIGGMLVAEAFEHTPGIQTASLSMYLKTNLFLFLFALGFYLLLRLLDIDLLWSVPIAK
Mouse                  LGVIGGMLVAEAFEHTPGVHMASLSVYLKTNVFLFLFALGFYLLLRLFGIDLLWSVPIAK
Minke whale            LGVIGGMLVAEAFEHTPRIQTASLSTYLKTNLFLFLFALGFYLLLRLLDIDLLWSVPIAK
Chimpanzee             LGVIGGMLVAEAFEHTPGIQTASLGTYLKTNLFLFLFALGFYLLLRVLNIDLLWSVPIAK
Water buffalo          LGVIGGVLVAEVFEYTPGIQTASLSIYLKTNLFLFLFALGFYLLLRRLDIDLLWSVPIAK
Green monkey           PGVIGGMLVAEAFEHTPDIQTASLGTYLKTSLFLFLFALGFYLLLRLLNIDLLWSVPIAK
Polar bear             LGVFGGMLVAEAFEHTPGIQTASLSTYLKTNLFLFLFALGFYLLLRLLDIDLLWSVPIAK
Orangutan              LGVIGGMLVAEAFEHTPGIQTASLGTYLKTNLFLFLFALGFYLLLRLLNIDLLWSVSIAK
Marmoset               LGVIGGMLVAEAFEHTPSIQTASLGTYLKTNLFLFLSALGFYLLLRLLSIDLLWSVTIAK
Sheep                  LGVIGGVLVAEVFEYTPGIQTASLSTYLKTNLFLFLFALGFYLLLRLLDIDLLWSVPIAK
Rabbit                 LGVIGGMLVAEAFEHTPGIQTASLSTYLKTNLFLFLFALGFYLLLRLLDIDLLWSVPIAK
Naked mole-rat         LGVIGGMLVAEAFEHTPSLHMASLSTYLKTNLFLFLFALGFYLLLRLLNIDLLWSVPIAK
Chinchilla             LGVIGGMLVAEAFEHTPGIHTASLSTYLKTNLFLFLFALGFYLLLRLLNIDLLWSVPIAK
Ferret                 LGVFGGMLVAEAFEHTSGIQTASLSTYLKTSLFLFLFALGFYLLLRLLDIDLLWSLPIAK
Elephant               LGVIGGMLVAEAFEHTPGIQTASLSTYLKTNLFLFLFALGFYLLLSLLNIDLLWSVPIAK
Capra                  LGVIGGVLVAEVFEYTPGIQTASLSTYLKTNLFLFLFALGFYLLLRLLDIDLLWSVPIAK
Pacific walrus         LGVFGGMLVAEAFEHTPGIQTAGLSTYLKTNLFLFLFALGFYLLLRLFDIDLLWSLPIAK
Weddell seal           LGVFGGMLVAEAFEHTPGIQTASLSTYLKTNLFLFLFALGFYLLLRLFDIDLLWSLPIAK
Gibbon                 LGVIGGMLVAEAFEHTPGIQTASLGTYLKTNLFLFLFALGFYLLLRLLNIDLLWSVPIAK
Tenrec                 LGVIGGMLVAEAFEHTRAIQTASLTTYLKTNLFLFLFALGFYLLLSLLDIDLLWSVPIAK
Shrew                  LGVIGGMLVAEAFEYTPDIQTASLATYLKTNLFLFLFALCFYLLLRVLDIDLLWSVPIAK
```

Horse	LGVIGGMLVAEAFEHTPGIQTASLSTYLKTNLFLFLFALGFYLLLRLLDIDLLWSVPIAK
Guinea pig	LGVIGGMLVAEAFEHTPGIHTASLSTYLKTNLFLFLFALGFYLLLRLLDIDLLWSVPIAK
Brush-tailed rat	LGVIGGMLVAEAFEHTPGIHTASLSTYLKTNLFLFLFALGFYLLLRLLDIDLLWSVPIAK
Aardvark	LGVIGGMLVAEAFEHTPGIQTASLSTYLKTNLFLFLFALGFYLLLSLLDIDLLWSVPIAK
Dolphin	LGVIGGMLVAEAFEHTPRIQTASLSTYLKTNLFLFLFALGFYLLLRLLDIDLLWSVPIAK
Alpaca	LGVIGGMLVAEASEHTPSIQSASLSTYLKTNLFLFLFALGLYLLLGLLDIDLLWSVPIAK
Manatee	LGVIGGMLVAEAFEHTPGIQTASLSTYLKTNLFLFLFALGFYLLLSLLDIDLLWSVPIAK
Tree shrew	LGVIGGMLVAEAFEHTPGIQTASLSTYLKTNLFLFLFAYGFYLLLRLLDIDLLWSVPIAK
Baboon	PGVIGGMLVAEAFEHTPGIQTASLGTYLKTNLFLFLFALGFYLLLRLLNIDLLWSVPIAK
Rhesus macaque	PGVIGGMLVAEAFEHTPGIQTASLGTYLKTNLFLFLFALGFYLLLRLLNIDLLWSVPIAK
Cheetah	LGVIGGMLVAEAFEHTPGIQTASLSTYLKTNLFLFLFALGFYLLLRLLDIDLLWSVPIAK
Big brown bat	LGVIGGMLVAEAFEHTPGIQTASLSTYLKTNLFLFLFALGFYLLLRLLDIDLLWSVPIAK
Sperm whale	LGVIGGMLVAEAFEHTPRIQTASLSTYLKTNLFLFLFALGFYLLLRLLDIDLLWSVPIAK
Little brown bat	LGVIGGMLVAEAFEHTPGIQTASLSTYLKTNLFLFLFSLGFYLLLRLLDIDLLWSVPIAK
Cow	LGVIGGVLVAEIFEYTPGIQTASLSVYLKTNLFLFLFALGFYLLLRHLDIDLLWSVPIAK
Armadillo	LGVIGGMLVAEAFEYTPGIQTASLNMYLKTNLFLFLFALGFYLLLRLLDIDLLWSVPIAK
Sunda flying lemur	LGVIGGMLVAEAFEHTPGIQTASLSTYLKTNLFLFLFALGFYLLLRLLDIDLLWSVPIAK
Star-nosed mole	LGVIGGMVVAEVFQHASGIQTASLSTYLKTNLFLFLFALGFYLLLRLLDIDLLWSVPIAK
Bactrian camel	LGVIGGMLVAEASEHTPSIQSASLSTYLKTNLFLFLFALGLYLLLGLLDIDLLWSVPIAK
Gorilla	LGVIGGMLVAEAFEHTPGIQTASLGTYLKTNLFLFLFALGFYLLLRLLNIDLLWSVPIAK
Pig	LGVIGGMLVAEVFEHTPGIQTASLSTYLKTNLFLFLFALGLYLLLGLLDIDLLWSVPIAK
Plains bison	LGVIGGVLVAEVFEYTPGIQTASLSVYLKTNLFLFLFALGFYLLLRRLDIDLLWSVPIAK
Flying fox	LGVIGGMLVAEAFEHTPGIQTASLSTYLKTNLFLFLFALGFYLLLRLLDIDLLWSVPIAK
Golden hamster	LGVIGGMLVAEAFEHTPGILMASLNMYLKTNIFLFLFALGFYLLLRLLDIDLLWSVPIAK
Cape golden mole	LGVIGGMLVAEAFEHTPGIQTASLSTYLKTNLFLFLFALGFYLLLSLLDIDLLWSVPIAK
Cape elephant shrew	LGVIGGMLVAEAFEHTPGIQTASLSTYLKTNLFLFLFALGFYLLLSLLDIDLLWSVPIAK
Squirrel monkey	LGVIGGMLVAEAFEHTPGIQTASLGTYLKTDLFLFLFALGFYLLLRLLSIDLLWSVPIAK
Cat	LGVLGGMLVAEAFEHTPGIQTASLSTYLKTNLFLFLFALGFYLLLRLLDIDLLWSVPIAK
Bushbaby	LGVIGGMLVAEAFEHTPGIQTATLSMYLKTNLFLFLFALGFYLLLRLLNIDLLWSVPIAK
Killer whale	LGVIGGMLVAEAFEHTPRIQTASLSTYLKTNLFLFLFALGFYLLLRLLDIDLLWSVPIAK
Pika	LGVIGGMLVAEAFEHTPGIQTASLSTYLKTNLFLFLFALGFYLILRLLDIDLLWSVPIAK
Tiger	LGVIGGMLVAEAFEHTPGIQTASLSTYLKTNLFLFLFALGFYLLLRLLDIDLLWSVPIAK
Black flying-fox	LGVIGGMLVAEAFEHTPGIQTASLSTYLKTNLFLFLFALGFYLLLRLLDIDLLWSVPIAK
Panda	LGVFGGMLVAEAFEHTPGIQTASLSTYLKTNLFLFLFALGFYLLLRLFDIDLLWSVPIAK
Tibetan antelope	LGVIGGVLVAEVFEYTPGIQTASLSTYLKTNLFLFLFALGFYLLLRLLDIDLLWSVPIAK
Free-ranging baiji	LGVIGGMLVAEVFEHTPRIQTASLSTYLKTNLFLLLFALGFYLLLRLLDIDLLWSVPVAK
Lesser jerboa	LGVIGGMLVAEAFEYTPGILTASLSLYLKTNLFLFLFALGFYLLLRMLDIDLLWSVPIAK
Dog	LGVFGGMLVAEAFEHTPGIQTASLSTYLKTNLFLFLFALGFYLLLRLLDIDLLWSVPIAK
White rhinoceros	LGVIGGMLVAEAFEHTPGIQTASLSTYLKTNLFLFLFALGFYLLLRLLDIDLLWSVPVAK
White donkey	LGVIGGMLVAEAFEHTPGIQTASLSTYLKTNLFLFLFALGFYLLLRLLDIDLLWSVPIAK
Crab-eating macaque	PGVIGGMLVAEAFEHTPGIQTASLGTYLKTNLFLFLFALGFYLLLRLLNIDLLWSVPIAK
Chinese hamster	LGIIGGMLVAEAFEHTPGIHMANLNVYLKTNIFLFLFALGFYLLLRLLDIDLLWSVPIAK

	297 298
Human	KWCANPDWIHIDTTPFAGLVRNLGVLFGLGFAINSEMFLLSCRGGNNYTLSFRLLCALTS
Squirrel	KWCANPDWIHIDTTPFAGLVRNLGVLFGLGFAINSEMFLMSCRGENGYKPSFRLLCAITS
Mouse	KWCANPDWIHIDSTPFAGLVRNLGVLFGLGFAINSEMFLRSCQGENGTKPSFRLLCALTS
Minke whale	KWCANPDWIHIDTTPFAGLVRNLGVLFGWGFAINSEMFLRSCRGENGYRLSFRLLCTLAS
Chimpanzee	KWCANPEWIHIDTTPFAGLVRNLGVLFGLGFAINSEMFLXXXRGGNNYTLSFRLLCALTS
Water buffalo	KWCANPDWIHIDTTPFAGLVRNLGVLFGLGFAINSEMFLRSYRGENGYRLSFRLLCALVS
Green monkey	KWCANPDWIHIDTAPFAGLVRNLGVLFGLGFAVNSEMFLPSCRGGNSYTLSFRLLCVLTS
Polar bear	KWCANPDWIHIDTTPFAGLVRNLGVLFGLGFAINSEMFLRSCRGENGYKLSFRLLCAVTS
Orangutan	KWCANPDWIHIDTTPFAGLVRNLGVLFGLGFAINSEMFLXXXRGGNSYTLSFRLLCAFTS
Marmoset	KWCANPDWIHIDTTPFAVLVRNLGVLFGLGFAINSEMILMSCRGGKSYTLSFRLLCALTS
Sheep	KWCVNPDWIHIDTTPFAGLVRNLGVLFGLGFAINSEMFLRSCRGENGYRLSFRLLCTLAS
Rabbit	KWCANPDWIHIDTTPFAGLVRNLGVLFGLGFAINSEMFLXXXRGENGYKLSFRVLCAMTS
Naked mole-rat	KWCANPDWIHIDTTPFAGLVRNLGVFFGLGFAINSEMFLLSCRGENGYKLSFRVLCSMTS
Chinchilla	KWCANPDWIHIDTTPFAGLVRNLGVLFGLGFAINSEMFLLSCRGENSYKLSFRLLCVMTS
Ferret	KWCANPDWIHIDTTPFAGLVRNLGVLFGLGFAINSEMFLRSCRGENGYKLSFRLLCAVAS
Elephant	KWCANPDWIHIDTTPFAGLVRNLGVLFGLGFAINSEMFLLSCQGENGYKLSFRLLCVMTS
Capra	KWCANPDWIHIDTTPFAGLVRNLGVLFGLGFAINSEMFLRSCRGENGYRLSFRLLCALAS
Pacific walrus	KWCANPDWIHIDTTPFAGLVRNLGVLFGLGFAINSEMFLRSCRGENGYKLSFRLLCAVAS
Weddell seal	KWCANPDWIHIDTTPFAGLVRNLGVLFGLGFAINSEMFLRSCRGENGYKLSFRLLCTVAS
Gibbon	KWCANPDWIHIDTTPFAGLVRNLGVLFGLGFAINSEMFLXXXRGGNSYTLSFRLLCALTS
Tenrec	KWCANPDWVHIDTTPFVGLVRNLGVLFGLGFAIHSEMFLKSCXGGNGCKLSFRLLSVMTS
Shrew	KWCANPEWIHIDTTPFAGLVRNLGVLVGLGLAINSDMFLRSCRGENGYKLNFRLLCIVAS

Horse	KWCANPDWIHIDTTPFAGLVRNLGVLFGLGFAINSEMFLQSCRGENRYKLSFRLLCAAAS
Guinea pig	KWCANPDWIHIDTTPFAGLVRNLGVLFGLGFAINSDMFLLSCRGENSYKLSFRLLCVMTS
Brush-tailed rat	KWCANPDWIHIDTTPFAGLVRNLGVLFGLGFAINSEMFLLSCRGENSYKLSFRLLCVTTS
Aardvark	KWCANPDWIHIDTTPFAGLVRNLGVLFGLGFAINSEMFLTSCQGENGYKPSFRLLCMMTS
Dolphin	KWCANPDWIHIDTTPFAGLVRNLGVLFGLGFAINSEMFLRSCRGENGYRLSFRLLCTLAS
Alpaca	KWCANPDWIHIDTTPFAGLVRNLGVLFGLGFAIHSEMFLRSCRGETGYGLSFRLLCAGAS
Manatee	KWCANPDWIHIDTTPFAGLVRNLGVLFGLGFAINSEMFLMSCQGENGYKLSFRMLCAITS
Tree shrew	KWCANPDWIHIDTTPFAGLVRNLGVLFGLGFAINSEMFLMSCKGENGYKLNFRLLCTMTS
Baboon	KWCANPDWIHIDTTPFAGLVRNLGVLFGLGFAVNSEMLLLSCXGGYSYTLSFRLLCALTS
Rhesus macaque	KWCANPDWIHIDTTPFAGLVRNLGVLFGLGFAVNSEMFLXXXRGGYSYTLSFRLLCALTS
Cheetah	KWCANPDWIHIDTTPFAGLMRNLGVLFGLGFAINSEMFLRSCRGENGYKLSFRLLCAVVS
Big brown bat	KWCANPDWIHIDTTPFAGLVRNLGVLFGLGFAINSEMFLRSCRGENGYKLSFRLLCVVAS
Sperm whale	KWCANPDWIHIDTTPFAGLVRNLGVLFGLGFAINSEMFLRSCRGENGYRLSFRLLCTLAS
Little brown bat	KWCANPDWIHIDTTPFAGLVRNLGVLFGLGFAINSEMFLRSCRGENGYKLSFRLLCVVAS
Cow	KWCANPDWIHIDTTPFAGLVRNLGVLFGLGFAINSEMFLRSCRGENGYGLSFRLLCALVS
Armadillo	KWCANPDWIHIDTTPFAGLVRNLGVLFGLGFAVNSEMFLMSCRGEKGYQPSFRLLCAMTS
Sunda flying lemur	KWCANPDWIHIDTTPFAGLVRNLGVLFGLGFAINSDMFLTSCRGENGYKLSFRLLCTMTS
Star-nosed mole	KWCADPDWIHIDTTPFAGLVRNLGTLFGLGFAINSEMFLRSCRGENSYKLSFRLLCVVAS
Bactrian camel	KWCANPDWIHIDTTPFAGLVRNLGVLFGLGFAIHSEMFLRSCGGETGYGLSFRLLCAGAS
Gorilla	KWCANPDWIHIDTTPFAGLVRNLGVLFGLGFAINSEMFLXXXRGGNNYTLSFRLLCALTS
Pig	KWCANPEWIHIDTTPFAGLVRNLGVLFGLGFAINSEMFLRSCRGENGYRLSFRLLCTMAS
Plains bison	KWCANPDWIHIDTTPFAGLVRNLGVLFGLGFAINSEMFLRSCQGENGYRLSFRLLCALVS
Flying fox	KWCANPDWIHIDTTPFAGLVRNLGVLFGLGFAINSEMFLRSCRGENGYKLSFRLLCILAS
Golden hamster	KWCANPDWIHIDSTPFAGLVRNLGVLLGLGFAINSKMFLKSCQGENGCKLSFRLLCAKAS
Cape golden mole	KWCANPDWIHIDTTPFAGLMRNLGVLFGLGFAINSKMFLMSCKGENGYKLSFRLLCVMTS
Cape elephant shrew	KWCANPDWIHIDTTPFAGLVRNLGVLFGLGFAINSKMFLMSCQGENGYKLSFRLLCVMTS
Squirrel monkey	KWCANPDWIHIDTTPFAGLVRNLGVLFGLGFAINSKMFLMSCRGGRSYTLSFRLLCALTS
Cat	KWCANPDWIHIDTTPFAGLMRNLGVLFGLGFAINSEMFLRSCRGENGYKLSFRLLCAVVS
Bushbaby	KWCANPDWIHIDTTPFAGLVRNLGVLFGLGFAINSEMFLMSCRGENSCKPSFRLLCATTS
Killer whale	KWCANPDWIHIDTTPFAGLVRNLGVLFGLGFAINSEMFLRSCRGENGYRLSFRLLCTLAS
Pika	KWCANPDWIHIDTTPFAGLVRNLGVLFGLGFAINSEMFQXXXRGENGYKLSFRVLCAITS
Tiger	KWCANPDWIHIDTTPFAGLMRNLGVLFGLGFAINSEMFLRSCRGENGYKLSFRLLCAVVS
Black flying-fox	KWCANPDWIHIDTTPFAGLVRNLGVLFGLGFAINSEMFLRSCRGENGYKLSFRLLCVLAS
Panda	KWCANPDWIHIDTTPFAGLVRNLGVLFGLGFAINSEMFLRSCRGENGYKLSFRLLCAVTS
Tibetan antelope	KWCANPDWIHIDTTPFAGLVRNLGVLFGLGFAINSEMFLRSCRGENGYRLSFRLLCALAS
Free-ranging baiji	KWCANPDWIHIDTTPFAGLVRNLGVLFGLGFAINSEMFLRSCRGGNGYRLSFRLLCTLAS
Lesser jerboa	KWCANPDWIHMDTTPFAGLVRNLGVLFGLGFAINSDMFLXXXRGENGYKPRFRLLCAATS
Dog	KWCANPDWIHIDTTPFAGLVRNLGVLFGLGFAINSEMFLRSCRGENGYKLSFRLLCAGAS
White rhinoceros	KWCANPDWIHIDTTPFAGLVRNLGVFFGLGFAINSEMFLQSCRGENRYKLSFRLLCAVAS
White donkey	KWCANPDWIHIDTTPFAGLVRNLGVLFGLGFAINSEMFLQSCRGENRYKLSFRLLCAAAS
Crab-eating macaque	KWCANPDWIHIDTTPFAGLVRNLGVLFGLGFAVNSEMFLXXXRGGYSYTLSFRLLCALTS
Chinese hamster	KWCANPDWIHIDSTPFAGLVRNLGVLFGLGFAINSEMFLKSCQGENGDKLSFQLLCAITS

	316 324 342 351
Human	LTILQLYHFLQIPTHEEHLFYVLSFCKSASIPLTVVAFIPYCVHMLMKQSGKKSQ
Squirrel	LTTLQLYHFIKIPTHTEHLFYMLSFCKSASIPLTVVALIPYCIHMLMKPSEKKMK
Mouse	LTTMQLYRFIKIPTHAEPLFYLLSFCKSASIPLMVVALIPYCVHMLMRPGDKKTK
Minke whale	LTTLQLYHFIKIPTHTEYLFYVLSFCKSASIPLTVVALIPYCIHMLMKPSEKKIH
Chimpanzee	LTILQLYHFLQIPTHEEHLFYVLSFCKSASIPLTVVAFIPYCVHMLMKQSGKKSQ
Water buffalo	LTTLQLYHFIKIPTDTEYLFYVLSFCKSASIPLTVVALIPYCIHMLMKPSAKKLN
Green monkey	LTTLXLYHFLQILTQEEHLFYVLSFCKSASIPLTVVTFIPYCVHMLMKQNRKKIQ
Polar bear	LITLQLYHFIKIPTHAEHLFYILSFCKSASIPLTVVALIPYCIHMLMKPSEKKIN
Orangutan	LTTLQLYHFLQIPTHEEHLFYVLSFCKSASIPLTVVAFIPYCVHMLMKQSGKKIQ
Marmoset	LTTLELYHFLQIPTHEEHLFYVLSFCKSASIPLTVVSLIPYCVRMLLKQSRKKIQ
Sheep	LTTLQLYHFIKIPTDTEYLFYVLSFCKSASIPLTVVALIPYCIHMLMKPSAKKLN
Rabbit	LTTLQLYHFVKIPTHTEHLFYVLSFCKSASIPLTVVALIPYCIHMLMKPSEKKTN
Naked mole-rat	LATLQLYHFIKIPTHTEHLFYMLSFCKSASIPLTVVALIPYCIHMLMKPNEKKFK
Chinchilla	LTTLQLYHFIKIPTHTEHLFYMLSFCKSASIPLTVVALIPYCIHMLMQPREKKIK
Ferret	LITLQLYHFIKIPTHAEHLFYILSFCKSASIPLTVVALIPYCIHVLMKPSEKKIN
Elephant	LTTLQLYHFIKIPTHTEHLFYVLSFCKSASIPLTVVALIPYCIHTLMKPSEKKTK
Capra	LTTLQLYHFIKIPTDTEYLFYVLSFCKSASIPLTVVALIPYCIHMLMKPSAKKLN
Pacific walrus	LITLQLYHFIKIPTHAEHLFYVLSFCKSASIPLTVVALIPYCIHVLMKPSEKKIN
Weddell seal	LITLQLYHFIKIPTHAEHLFYVLSFCKSASIPLTVVALIPYCIHVLMKPREKKIN
Gibbon	LTTLQLYHFLQIPTHEEHLFYVLSFCKSASIPLTVVAFIPYCVHILMKQSRKKIQ
Tenrec	LTVLPLYQSTQIPTHIEHLFCVFSFCKSASIPLAVVALIPYCIHTLMKPSDKKIN
Shrew	LIILQLYHFIKIPTHPEHLFYVLSFCKSASIPLTVVALIPYCIHMLIEXXNGKDD

Horse	LTILQLYHFIKIPTHSEHLFYVLSFCKSACIPLAVVAVIPYCIHMLMKPSEKKIN
Guinea pig	LTTLQLYHFIKIPTHTEHLFYILSFVKSASIPLTVVALIPYCIHMLMQPREKKIK
Brush-tailed rat	LTTLQLYHFIKIPTHTEHLFYMLSFCKSASIPLTVVALIPYCIHMLMQSREKKIK
Aardvark	LTTLQLYHLVKIPTHAEHLFYALSFCKSASIPLTVVAFIPYCIHRVMKPSEKKNN
Dolphin	LTTLQLYHFVKIPTHTEYLFYMLSFCKSASIPLTVVALIPYCIHMLMKPSEKKIN
Alpaca	LTTLQLYHFIKIPTHAEYLFYVLSFCKSASIPLTVVAFIPYCMHILMKPREKKIN
Manatee	LTTLQLYRFIKIPTHTEHLFYVLSFCKSASIPLTVVAFIPYCIHMLMKPSEKKIN
Tree shrew	LTTLQLYHFIKIPTHKEHLFYVLSFCKSASIPLTVVALVPYCIHMFMKPSEKKMK
Baboon	LTTQQLYHFLQILTQEEHLFYVLSFCKTASIPLTVVTFIPYCVHMLMKQNGKKIQ
Rhesus macaque	LTTLHLYHFLQIPTQEEHLFYVLSFCKSASIPLTLVTFIPYYVHMLMKQNGKKIQ
Cheetah	LITLQLYHFIKIPTHAEHLFYVLSFCKSASIPLTVVALIPYCIHMLMKPSEKKIN
Big brown bat	LTTLQLYHFIKIPTHTEHLFYVLSFCKSASIPLTVVALIPYCIHVLMKPSEKKID
Sperm whale	LTTLQLYHFIKIPTHTEYLFYVLSFCKSASIPLTVVALIPYCIHMLMKPSEKKIN
Little brown bat	LTILQLYHFIKIPTHTEHLFYVLSFCKSASIPLTVVALIPYCIHVLMTPSEKKID
Cow	LTTLQLYHFIKIPTDTEYLFYVLSFCKSASIPLTVVALIPYCIHMLMKPSAKKLN
Armadillo	LTTLQLYHFIKIPTHVEHLFYVLSFCKSASIPLTVVALIPYCIHMLMKPSGKKIP
Sunda flying lemur	LTTLQLYHYVKIPTHAEHLFYVLSFCKSASIPLAVVALIPYCIHMLMKPSEKKIK
Star-nosed mole	LATLQLYHFIKLPTHTEHLFYVLSFCKSASIPLTVVALIPYCIHMLMKPREKKID
Bactrian camel	LTTLQLYHFIKIPTHTEYLFYVLSFCKSASIPLTVVALIPYCMHILMKPREKKIN
Gorilla	LTILQLYHFLQIPTHEEHLFYVLSFCKSASIPLTVVAFIPYCVHMLMKQSGKKIQ
Pig	LTTLQLYHFIKIPTQAEYLFYVLSFCKSASIPLTVVALIPYCIHALMKPSKKKIH
Plains bison	LTTLQLYHFIKIPTDTEYLFYVLSFCKSASIPLTVVALIPYCIHMLMKPSDKKLN
Flying fox	LTTLQLYHFIKIPTHAEHLFYVLSFCKSASIPLTVVALIPYCIHMLMKPSAKKVN
Golden hamster	LAALQLHPFIKIPTHREPLFYLLSFCKSAPIPLTVVALIPYCVRMLLRTSEKKTK
Cape golden mole	LTTLQLYHFIKIPTHTERLFYVLSFCKSASIPLTVVALIPYCIHMLMKPSEKKIH
Cape elephant shrew	LTTLQLYHFIKIPTHTEHLFYVLSFCKSASIPLTVVALIPYCIHVLMKPNEKKID
Squirrel monkey	LTTLQLYHFLQIPTQEEHLFYVLSFCKSASIPLTVVALIPYCVHMLLKQSGKKIQ
Cat	LITLQLYHFIKIPTHAEHLFYVLSFCKSASIPLTVVALIPYCIHMLMKPSEKKIN
Bushbaby	LATLQLYHFIEIPTQQEHLFYVLSFCKSASIPLAVVALIPYCIHMLMKPSEKKIK
Killer whale	LTTLQLYHFVKIPTHTEYLFYMLSFCKSASIPLTVVALIPYCIHMLMKPSEKKIN
Pika	LTTLQLFHFVKIPTHTEHLFYVLSFCKSASIPLTVVALIPYCIHMLMKPNEKKTN
Tiger	LITLQLYHFIKIPTHAEHLFYVLSFCKSASIPLTVVALIPYCIHMLMKPGEKKIN
Black flying-fox	LTTLQLYHFIKIPTHAEHLFYVLSFCKSASIPLTVVALIPYCIHMLMKPSAKKVN
Panda	LITLQLYHFIKIPTHAEHLFYILSFCKSASIPLTVVALIPYCIHMLMKPSEKKIN
Tibetan antelope	LTTLQLYHFIKIPTDTEYLFYVLSFCKSASIPLTVVALIPYCIHMLMKPSAKKLN
Free-ranging baiji	LTTLQLYHFVKIPTHTEYLFYVLSFCKSASIPLTVVALIPYCIHMLMKPTEKKIN
Lesser jerboa	VTALQLYRLVHVPTHTEHLFYVLSFCKSASIPLTVVALIPYCVHTAMQPRGKKIN
Dog	LMTLQLYHFIKIPTHAEHLFYVLSFCKSASIPLTVVALIPYCIHMLMKPSEKKIN
White rhinoceros	LTLLQLYHFIKIPTHSEHLFYVLSFCKSACIPLAVVAVIPYCIHMLMKPSEKKIN
White donkey	LTILQLYHFIKIPTHSEHLFYVLSFCKSACIPLAVVAVIPYCIHMLMKPSEKKIN
Crab-eating macaque	LTTLHLYHFLQIPTQEEHLFYVLSFCKSASIPLTLVTFIPYYVHMLMKQNGKKIQ
Chinese hamster	LATLQLYHFIKIPTHREPLFYLLSFCKSASIPLTVVALIPYCVHMFMRASEKKTK
